# NEMO/NF-κB signaling functions as a double-edged sword in PanIN formation versus progression to pancreatic cancer

**DOI:** 10.1186/s12943-024-01989-x

**Published:** 2024-05-16

**Authors:** Miltiadis Tsesmelis, Ulrike F. G. Büttner, Melanie Gerstenlauer, Uta Manfras, Konstantinos Tsesmelis, Ziwei Du, Nadine Sperb, Stephanie Ellen Weissinger, Peter Möller, Thomas F. E. Barth, Harald J. Maier, Lap Kwan Chan, Thomas Wirth

**Affiliations:** 1 Institute of Physiological Chemistry, University of Ulm, Meyerhofstrasse, 89081 Ulm, Baden-Württemberg Germany; 2 Alb Fils Kliniken Göppingen, 73035 Göppingen, Baden-Württemberg Germany; 3 Institute of Pathology, University of Ulm, 89081 Ulm, Baden-Württemberg Germany; 4 Novartis Pharma, 4056 Basel, AG Switzerland; 5 Department of Pathology and Molecular Pathology, University Hospital of Zurich, 8091 Zurich, Switzerland; 6 Institute of Molecular Cancer Research, University of Zurich, 8057 Zurich, Switzerland

**Keywords:** PDAC, PanINs, NEMO, NF-κB, Senescence, Cerulein, Pancreatitis

## Abstract

**Background:**

Pancreatic ductal adenocarcinoma (PDAC) is marked by a dismal survival rate, lacking effective therapeutics due to its aggressive growth, late-stage diagnosis, and chemotherapy resistance. Despite debates on NF-κB targeting for PDAC treatment, no successful approach has emerged.

**Methods:**

To elucidate the role of NF-κB, we ablated NF-κB essential modulator (NEMO), critical for conventional NF-κB signaling, in the pancreata of mice that develop precancerous lesions (KC mouse model). Secretagogue-induced pancreatitis by cerulein injections was utilized to promote inflammation and accelerate PDAC development.

**Results:**

NEMO deletion reduced fibrosis and inflammation in young KC mice, resulting in fewer pancreatic intraepithelial neoplasias (PanINs) at later stages. Paradoxically, however, NEMO deletion accelerated the progression of these fewer PanINs to PDAC and reduced median lifespan. Further, analysis of tissue microarrays from human PDAC sections highlighted the correlation between reduced NEMO expression in neoplastic cells and poorer prognosis, supporting our observation in mice. Mechanistically, NEMO deletion impeded oncogene-induced senescence (OIS), which is normally active in low-grade PanINs. This blockage resulted in fewer senescence-associated secretory phenotype (SASP) factors, reducing inflammation. However, blocked OIS fostered replication stress and DNA damage accumulation which accelerated PanIN progression to PDAC. Finally, treatment with the DNA damage-inducing reagent etoposide resulted in elevated cell death in NEMO-ablated PDAC cells compared to their NEMO-competent counterparts, indicative of a synthetic lethality paradigm.

**Conclusions:**

NEMO exhibited both oncogenic and tumor-suppressive properties during PDAC development. Caution is suggested in therapeutic interventions targeting NF-κB, which may be detrimental during PanIN progression but beneficial post-PDAC development.

**Graphical Abstract:**

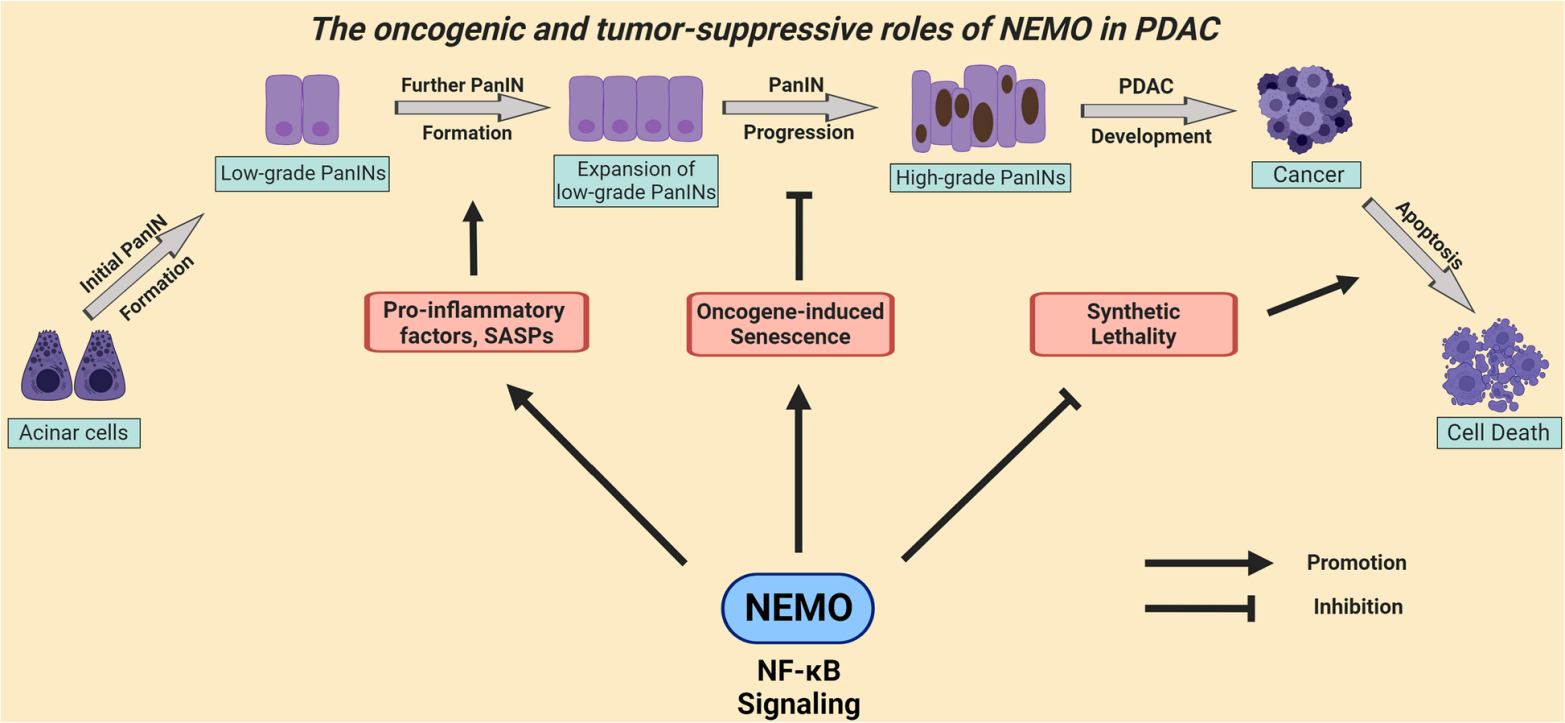

**Supplementary Information:**

The online version contains supplementary material available at 10.1186/s12943-024-01989-x.

## Ιntroduction

In 2022, pancreatic cancer ranked 3^rd^ for most cancer-related deaths in the USA, with a 5-year survival rate of 10%, and is predicted to rise to 2^nd^ by 2030 [1, 2]. Pancreatic ductal adenocarcinoma (PDAC) is the most common form of pancreatic cancer, originating mainly from pancreatic intraepithelial neoplasias (PanINs) which are categorized as low-grade or high-grade [3]. Common mutations in low-grade PanINs lead to KRAS proto-oncogene activation, detected in 95% of PDAC cases [3]. Finally, chronic pancreatitis significantly raises PDAC risk, with up to a 16-fold likelihood increase [4].

Although active NF-κB can be detected in 70% of PDAC cases, its role is not completely understood [5]. NF-κB is a dimeric transcription factor with different combinations of NFKB1/p50, NFKB2/p52, RelA/p65, RelB and c-Rel [5]. The prototypical dimer p50:p65 regulates the conventional NF-κB pathway. Under normal conditions, p50:p65 remains inactive in the cytoplasm, bounding to its inhibitor, IκBα. However, diverse stimuli can activate the IκB kinase (IKK) complex, comprising IKK1, IKK2, and NF-κB essential modulator (NEMO). Activated IKK complex phosphorylates IκBα, prompting its proteasomal degradation and facilitating NF-κB's nuclear translocation [5].

Notably, a link between constitutive KRAS activity and NF-κB activation has been described. In a murine-mutant KRAS model, oncogenic KRAS activated the activator protein-1 (AP-1) complex, which induced the expression of IL-1α, activated NF-κB and promoted the development of PDAC, while knockout of IKK2 blocked PDAC development [6]. Intriguingly, the role of NF-κB in the pancreas is heavily context dependent. For instance, our previous work revealed that in chronic pancreatitis, NEMO deletion sustained inflammation and fibrosis, inhibited acinar cell proliferation, and enhanced acinar atrophy and ADM formation [7]. In contrast, NEMO deletion in a murine mutant KRAS-driven model notably reduced pro-inflammatory cytokine/chemokine expression, ameliorated the infiltration of immune cells and markedly reduced PanIN numbers [8].

In the current study, we investigated the prevailing function of NF-κB using the well-established Pdx1-Cre;KRAS^G12D^ (KC) mouse model and subjected the mice to cerulein-induced pancreatitis to further support the development of PDAC [9]. Pancreata were analyzed at four time points: i) at 8 weeks, where the short-term effect of cerulein was evaluated, ii) at 6 months, iii) at 10 months, shortly before the median survival of the poorest survival group, and iv) at their humane endpoint (HEP) (Supplementary Figure S1A). NEMO deletion reduced the immune and fibrotic reactions but did not affect the development of PanINs at a young age. In contrast, although NEMO deletion strongly reduced the formation of PanINs at the age of 10 months, it accelerated the progression of low-grade PanINs toward high-grade PanINs and PDAC. This study reports the surprising finding that NEMO exerted a dual function in PanIN formation and PanIN progression. To our knowledge, this is the first description of a novel function of NEMO in PDAC development, in contrast to the previously reported findings involving IKK2 and RelA [10, 11].

## Methods

### Patient samples

Patient consent was obtained and adhered to the regulations of German legislation and publicly available data of TCGA for the survival analysis of patients from University Hospital Ulm and Human Protein Atlas (HPA), respectively. Adequate representation of male and female sex was ensured according to the SAGER guidelines. University Hospital Ulm cohort has been previously published [12]. Patient tissue sections with 10% or less of tissue covered by PDAC were excluded from the study. Patients’ age ranged between 56.4 and 81.5 years. These patients developed well, moderately, or poorly-differentiated pancreatic adenocarcinoma. Therapy was administered according to national guidelines [13].

### Mice

Mouse models, with C57BL/6J background expressing the Cre recombinase under the Pdx1 promoter [14], were crossed with mice carrying one *LSL-Kras*^*G12D*^ allele [15] and/or floxed *Ikbkg* alleles [16]. Both male and female mice were used in the experiments. The experimental unit was single animals. In case male mice were used to study the effect of NEMO deletion, only one *Ikbkg* allele was floxed since *Ikbkg* is X-linked [16]. Mice were analyzed at their 8^th^ week of age, 6^th^ month of age, 10^th^ month of age, or when they were in a moribund condition and had reached their humane end-point (HEP).

### Recombination efficiency

To calculate the recombination efficiency, we first examined the percentage of a-amylase^+^ cells having at least one *Ikbkg* signal in saline-injected 8-week-old KC and KNeC mice using Basescope. We then calculated the efficiency through the following formula:

$${\text{Recombination efficiency}}=100\times\left(1-\%\;{\text{of Ikbkg}}+{\text{amylase}}+{\text{cells in KNeC}}\,\big{/}\,\%\;{\text{of Ikbkg}}+{\text{amylase}}+{\text{cells in KC}}\right)$$

### Cerulein administration

Cerulein powder (Bachem #H-3220.0005) was dissolved in sterile ampuwa water (Fresenius Kabi #1080181) at a concentration of 0.5 mg/ml and afterward diluted 1:50 in NaCl 0,9% (Fresenius Kabi #PZN06605514) to prepare the working solution. Mice were injected intraperitoneally with the working solution, which was 50 μg cerulein/kg, at hourly intervals. Mice were injected for three days in total. The first injection day was when they reached their 6^th^ week of age and the mice were injected 6 times. The second injection day was 2 days after the first injection day and the mice were injected once with cerulein. The third injection day was 4 days after the first injection day and the mice were injected once with cerulein. Control groups were injected with NaCl 0,9% solution. Experiments and analyses of the mouse tissues were performed in the corresponding facilities in Ulm University.

### ADM, PanIN and cancer grading

Categorization of ADMs and PanINs and cancer grading was performed by our experienced pathologist team [3, 17]. In short, ADMs share traits of both acinar cells and ductal cells and no mucin accumulation. In contrast, PanINs do not share any acinar cell characteristics and are all characterized by mucin accumulation. Specifically, low-grade PanINs (PanIN1A, PanIN1B) are composed of columnar epithelial cells with basally oriented uniform and round nuclei, while high-grade PanINs (PanIN2, PanIN3) have more nuclear changes including loss of nuclear polarity, nuclear crowding and form papillae and cribriform structures. Based on these traits, we characterized the type of the lesions. In case a structure was composed of different-grade cells, the structure was always categorized according to the highest grade of cell type appearing in it (ADM —> low-grade PanIN —> high-grade PanIN —> Cancer).

### RNA extraction, cDNA synthesis and qRT-PCR

Isolated tissue was shortly washed in cold Hank’s balanced salt solution (HBSS), snap-frozen in liquid nitrogen and pulverized. mRNA was extracted using the RNeasy Mini Kit (Qiagen #74104). cDNA was synthesized with Transcriptor High Fidelity cDNA Synthesis Kit (Roche #5081955001). qRT-PCR was performed in Lightcycler 480 (Roche). For relative quantification, RPL13 was selected as the reference gene.

### Protein isolation, cytokine array and western blot

Tissue was shortly washed in cold HBSS, snap-frozen in liquid nitrogen and pulverized. Pancreatic powder was resuspended in buffer containing 4% sodium dodecyl sulfate (SDS), 100 mM Tris-HCl, phosphatase and protease inhibitors. Western blots were performed according to standard protocols. For cytokine array, pancreatic powder was resuspended in lysis buffer (Raybiotech, #AA-LYS-10ml) and applied to C3 and C4 membranes (Raybiotech, # AAM-CYT-1000-2). The membranes were then treated with biotinylated antibody, treated HRP-conjugated streptavidin, and treated with a chemiluminescence detection cocktail. The Western blot and cytokine array membranes were exposed either under x-ray films or using ChemiDoc MP Imaging System (Bio-Rad).

### Histology and immunostaining

For the analysis of mouse tissue, a longitudinal section including the head, body and tail of the pancreas was placed on a slide, supporting the evaluation of every compartment of the pancreas. From these sections, either the whole slice, 6 or 8 random pictures were captured and used for the analysis, according to the type of staining. For quantifying the recombination rate and the percentage of p65 localization to the nucleus, a minimum of 100 cells were evaluated. The number of captured fields for statistical analysis according to each staining is described in Table S1. Frozen or formalin-fixed paraffin-embedded (FFPE) tissues were used for immunostaining. A list of antibodies is presented in Supplementary Table S2. For Heidenhain’s azocarmine aniline blue stain (AZAN) staining, FFPE sections were stained with a kit for Heidenhain’s AZAN trichrome stain (Morphisto #12079) according to the manufacturer’s protocol. For TUNEL staining, FFPE sections were stained with In Situ Cell Death Detection Kit (Roche # 12156792910) according to the manufacturer’s protocol. For X-Gal staining, cryopreserved tissues were sectioned and stained with the Senescence β- Galactosidase Staining Kit (Cell signaling #9860S) according to the available protocol. Sections were counterstained with nuclear fast red, mounted with 70% glycerol (glycerol diluted in PBS 1X) and observed under the microscope. SBB staining has already been described in the past [18]. For X-gal and SBB stainings, a lesion was scored as positive when at least 10% of its cells were stained positive. For quantitative microscopy, the BZ-X810 microscope (Keyence) was used.

### Neutrophil presence in ductal structures

To evaluate the attraction of Ly6G^+^ cells (neutrophils) towards ADM/duct-like lesions or PanINs, we counted the number of Ly6G^+^ cells within a 20μm radius of an ADM/duct-like lesion or PanIN. When both an ADM/duct-like lesion and PanIN were within a 20μm radius, we selected the lesion nearest to the Ly6G^+^ cell.

### Basescope

Basescope probe against murine *Ikbkg* was designed to target nucleotides of exon 2 that are deleted in NEMO-flox mice. The thickness of each stained section was 3μm, including only a small part of each cell. Therefore, Basescope experiments were not used to detect all *Ikbkg* mRNA but to compare the presence of *Ikbkg* between NEMO-proficient and potential NEMO-deficient cells. The experiments were performed as instructed according to the manufacturer's protocol. Shortly, FFPE tissue slices were deparaffinized, treated with Hydrogen Peroxide for 10 minutes and treated with Target Retrieval Reagents for 10 minutes. Then, the main assay was applied with multiple hybridization steps for approximately 2 hours, the signal was detected and the slides were counterstained with hemalaun (Merck #109249).

### Isolation of acinar cells and TNF-α treatment

Pancreata from WT and NeC mice were dissociated with scissors in KRH buffer and were digested with collagenase P and trypsin inhibitor at 37°C for 20 minutes. The suspension was then filtered and centrifuged. Finally, the pellets were resuspended in RPMI medium (10% FCS) and treated with TNF-α (20 ng/ml) for 30 minutes.

### Nuclear extracts

Pancreatic tissue was incubated for 10 minutes in buffer A (10 mM HEPES, 1 mM PMSF, 1 mM DTT, 10 mM KCl, 1.5 mM MgCl_2_, protease inhibitors). Cells were lysed by aspirating using a 26-G needle. Nuclei were pelleted by centrifugation (5000 rpm, 4°C, 10 minutes), washed in buffer A, and incubated in buffer C (20 mM HEPES, 25% glycerol, 0.2 mM EDTA, 1.5 mM MgCl2, 1 mM PMSF, 1 mM DTT, 420 mM NaCl, protease inhibitors) for 1 hour. For nuclear extraction from acinar cells or murine embryonic fibroblasts, the same protocol was followed without the homogenization step.

### Electrophoretic mobility shift assay (EMSA)

Nuclear extracts (4μg) were incubated at room temperature for 30 minutes in binding buffer (50 mM KCl, 20 mM HEPES, 1 mM EDTA, 4% Ficoll, 1 mM DTT, supplemented with poly (dI/dC) and BSA) and radiolabeled double-stranded DNA probe containing an Ig-κ enhancer consensus NF-κB site. The DNA-protein complexes were separated on a native 4% polyacrylamide gel and visualized by autoradiography.

### Pancreatic-amylase enzymatic activity

Pulverized pancreatic tissue was resuspended in lysis buffer (Raybiotech, #AA-LYS-10ml) with protease/phosphatase inhibitors and the proteins were extracted. Solution of 1μg/μl was applied to pancreatic-amylase strips (Roche, #11126679202) and loaded to Reflotron® machine.

### Isolation of primary cancer cells and primary cell culture establishment

Pancreatic tumors were dissected with a scalper, cut into small pieces and incubated with collagenase D/HBSS (5mg/ml) (Roche #11088866001) for half an hour at 37°C. Collagenase D was then deactivated using culture medium DMEM (Gibco #41965-039), containing 10% fetal bovine serum (FBS) (Gibco #10270106). The cell suspension was successively applied to strainers of 100μm, 70μm and 40μm pores diameter. Then, the suspension was centrifuged, the pellet was resuspended in culture medium DMEM/F12 containing GlutaMAX (Gibco #31331028) and B-27 supplement (Gibco #17504-044) and finally seeded on ultra-low attachment plates (MilliporeSigma #CLS347124EA). Three days later, the cells were seeded to cell culture dishes (Greiner #664160) with DMEM (GIBCΟ #41965-039) containing 10% FBS and 1% L-glutamine (Gibco #25030-024). FibrOut 0.2% (VWR #10786-022) was added to the cell cultures for 6 days. Cells were then cultured for a maximum of 3 passages and were used for downstream applications.

### Etoposide treatment

To evaluate the induction of DNA damage, 3x10^4^ primary cancer cells were seeded at 24-well plates. The next day, the cells were treated with etoposide (MERCK #E1383) at a concentration of 500nM for 4 hours. The cells were then analyzed one- and two-days post-treatment and stained with γH2AX. Cells with at least 2 γH2AX foci in the nucleus were considered positive.

### FACS analysis

Fluorescence-activated cell sorting (FACS) was performed to evaluate cell death of cancer cells. In short, 10^6^ cells were washed with 500μl of 1x Annexin V binding buffer (0.1M HEPES pH 7.4, 1.4 M NaCl, 25 mM CaCl_2_) by centrifugation at 1200 rpm for 6 min. Cell pellet was then resuspended in 70 μl Annexin V binding buffer, 5 μl Annexin-V FITC (BD #556419) and 1.5 μl PI (1 mg/ml) and incubated for 15 minutes at room temperature in the dark. The samples were placed in the FACSCanto II device. Finally, samples were analyzed for their FITC and PI fluorescent signals using the FlowJo Software. To determine specific cell death, the calculated percentage of the living cells (PI^-^AnnexinV^-^ cells) was subtracted from the value of 100. This results in the number of "death" cells. By calculating these values for treated and non-treated cells, the following equation can be used to quantify specific cell death and compare the primary cell cultures: Specific cell death [%] = (Death_treated_ – Death_untreated_)/(100 – Death_untreated_) x100

### Laser capture microdissection (LCM)

Cryopreserved tissue was sectioned and placed in poly-L-lysine-coated membrane slides and air-dried for 5 minutes. Then, slides were incubated serially in 70% ethanol for 3 minutes, distilled water for 30 seconds, hemalum (Merck #109249, diluted 1:3 in distilled water) for one minute, distilled water for 30 seconds, 70% ethanol for 30 seconds, 90% ethanol for 30 seconds, 100% ethanol for 30 seconds and air-dried. Lesions were then excised and captured using the PALM Microbeam Rel. 4.9 with RoboMover microscope (Zeiss) and the PALMRobo v 4.9 software. RNA from isolated lesions was extracted with RNeasy Micro Kit (QIAGEN #74004). RNAse-free water was used in all steps (including dilution of ethanol) to avoid RNA degradation. Ethanol dilutions for the LCM preparation were stored at -20°C. RNAse-free water for the LCM preparation was stored at 4°C.

### RNA-Seq analysis

The DNBseq platform (BGI) was selected for the analysis of the RNA isolated from micro-dissected lesions (*N*=3/group). The total reads for each sample were between 99.43 million to 127.44 million. Filtering of low-quality reads was performed using the software SOAPnuke (v1.5.2). HISAT2 (v2.0.4) was used for the sequence alignment using the mouse Ensembl GRCm38 (mm10) as the reference genome [19]. The aligned reads were counted using the RSEM software. Differentially expressed genes (DEGs) between the 2 groups were detected using DEseq2 and visualized with gene set enrichment analysis (GSEA) and heatmap generation.

### Gene set enrichment analysis

RNAseq datasets from the Pdx1-Cre;KRAS^G12D^;NEMO^fl/fl^ (KNeC) and the Pdx1-Cre;KRAS^G12D^ (KC) groups were imported to GSEA v4.1.0 [20]. Analysis was performed using pre-ranked gene lists, which were generated according to the gene expression level (log2 fold change) of the KNeC to the KC group. The OI_SASP_FACTORS geneset was generated using 27 SASP genes that are upregulated in oncogene-induced senescence [21]. A list of genesets in GO_BP, GO_MF and KEGG databases enriched and depleted in KC vs KNeC lesions is provided in Additional File 1.

### Heatmap

To generate heatmaps, Rstudio and the library “gplots” were used. GOBP_DNA_Replication and GOBP_Recombinational_Repair genesets from the gene ontology database were used. The 30 most DEGs between KNeC and KC are visualized in the heatmaps for the abovementioned genesets.

### Patient categorization for survival analysis

For the survival analysis of human pancreatic cancer patients, the biobank of University Hospital Ulm and Human Protein Atlas (HPA) database were used to compare groups with respect to the expression of *IKBKG* (NEMO) [22, 23]. For HPA survival analysis, patients were classified into two groups (high and low expression) according to the fragments per kilobase of transcript per million (FPKM) values of RNAseq analysis for *IKBKG*. According to the HPA database, the selected expression cut-off for the two groups yields the maximal difference between the two groups concerning survival at the lowest log-rank *P*-value. For the survival analysis of University Hospital Ulm, CK7^+^ cells were evaluated for the presence of *IKBKG* in patient samples. CK7^+^ cells having at least one *IKBKG* Basescope signal were considered as *IKBKG*^+^CK7^+^. In case no *IKBKG* Basescope signal was observed, the cell was considered as *IKBKG*^-^CK7^+^. The percentage of *IKBKG*^+^CK7^+^ cells to the total number of CK7^+^ cells for each sample was calculated. We then divided our patient samples into two groups with equal number of patients (excluding censored values): high % of *IKBKG*^+^CK7^+^/ CK7^+^ cells (median: 31.2% *IKBKG*^+^CK7^+^/ CK7^+^ cells; range: 18.9% to 67.2%) and low % of *IKBKG*^+^CK7^+^/ CK7^+^ cells (median: 11.7% *IKBKG*^+^CK7^+^/ CK7^+^ cells; range: 4.8% to 17.6%). The survival of these two groups of patients was compared.

### Statistics

Statistical analyses were performed with Graphpad Prism v.8.4.3. Diagrams show arithmetic means and standard deviations. For the saline-injected WT, NeC, KC and KNeC groups, an effect size of 0.9601432, an α of 0.025 and a power of 0.8 was used. A number of 5 animals per group was selected. For the cerulein-injected WT, NeC, KC and KNeC groups, an effect size of 0.559017, an α of 0.025 and a power of 0.8 was used. A number of 12 animals per group was selected. The exact number of mice used was decided according to the downstream application/experiment performed. Blinding was not applicable, as we aimed to use all the possible animals with the desired genotypes after genotyping. However, stratified randomization strategy was used, in order to maintain the same distribution of both sexes in control groups and experimental groups. No criteria for including or excluding animals were used. No exclusion of animals was done. Student’s t-test was used for the comparison of 2 groups while one-way analysis of variance (ANOVA) with Tukey’s multiple comparison test was used for the comparison of 3 or more groups (*p <0.05, **p <0.01, ***p <0.001, ****p<0.0001). For the comparison of PDAC development between KC and KNeC mice, 1-tailed Fischer’s exact test was used (* *p* <0.05). For survival analysis, each group was examined by Kaplan-Meier survival estimators and the survival outcomes were compared using log-rank test (*p <0.05, **p <0.01) [12, 22, 23].

## Results

### NEMO deletion does not alter the number of precancerous lesions but reduces inflammation in cerulein-injected 8-week-old KC mice

**Fig. 1:**
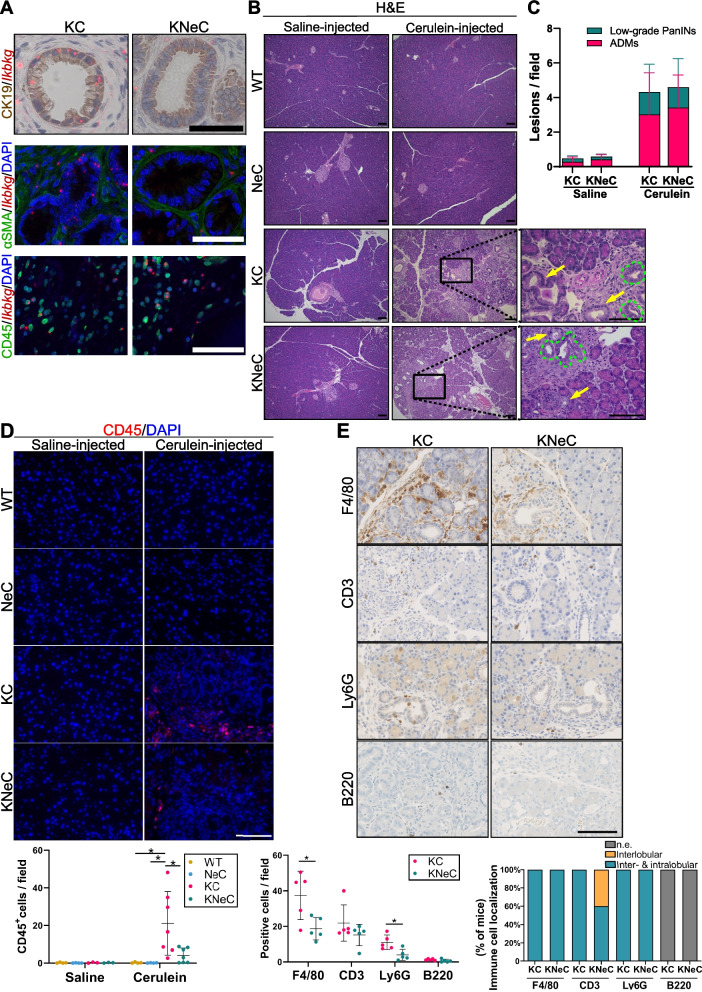
Pancreas-specific NEMO ablation reduces inflammation in cerulein-injected KC mice at the age of 8 weeks. **A** Visualization of *Ikbkg* transcripts in CK19^+^, αSMA^+^ and CD45^+^ cells in cerulein-injected KC and KNeC mice. Scalebars: 50μm, t=8 weeks. **B** H&E staining of pancreatic tissue from saline- or cerulein-injected mice. Area marked by green dash line: ADMs; Arrow: Low-grade PanIN. Scalebars: 100μms, t=8 weeks. **C** Quantification of lesions (ADMs, PanINs) of saline- or cerulein-injected KC and KNeC mice. Saline-injected groups: N≥3 mice/group, two-tailed Student’s t test; cerulein-injected groups: N≥7 mice/group, two-tailed Student’s t test; t=8 weeks. **D** Top: Visualization of immune cells by CD45 staining on pancreatic sections of saline- or cerulein-injected mice. Scalebar: 100μm. Bottom: Quantification of CD45^+^ cells. Cerulein-injected KC and KNeC groups: N≥7 mice/group, rest of groups: N≥3 mice/group; t=8 weeks. One-way ANOVA-Tukey for saline-injected groups; One-way ANOVA-Tukey for cerulein-injected groups. **E** Top: Visualization of immune cell sub-populations on pancreatic sections of cerulein-injected KC and KNeC mice. Scalebar: 100μm. Bottom: Quantification of immune sub-populations and localization of immune cells in each mouse. *N*=5 mice/group; t=8 weeks, n.e.: not examined. Two-tailed Student’s t test for positive cells/field; Two-tailed Fisher exact test for immune cell localization. Dot plot: Dots represent individual animals. Stack column: Values consist of individual mice. n.s.: *p* > 0.05; *p < 0.05; **p < 0.01; ***p < 0.001; ****p < 0.0001

To evaluate the role of NEMO in inflammation-driven precancerous lesions and PDAC development, we crossed mice expressing Cre recombinase under the Pdx1 promoter with mice carrying a Cre-induced *Kras*^*G12D*^ allele and/or floxed *Ikbkg* (NEMO) alleles (Table 1). We verified NEMO deletion in the pancreas by immunoblotting and examined compartment-specific deletion using Basescope (Figure 1A and Supplementary Figure S1B). The recombination efficiency of NEMO deletion in the exocrine compartment was 96.2% (Supplementary Figure S1C). To evaluate the functional consequences, we examined the activity of the canonical NF-κB pathway in acinar cells via EMSA. NEMO-deficient acinar cells demonstrated impaired pathway activation upon TNF-α stimulation, highlighting the crucial role of NEMO in canonical NF-κB signaling. (Supplementary Figure S1D).

**Table 1:** Nomenclature of the mouse models

**Mouse model**	**Genotype**
WT	---
NeC	Pdx1-Cre;NEMO^fl/fl^
KC	Pdx1-Cre;LSL-KRAS^G12D^
KNeC	Pdx1-Cre;LSL-KRAS^G12D^;NEMO^fl/fl^

Mice underwent three episodes of cerulein injections at 6 weeks to induce pancreatitis, while saline-injected mice served as controls. At 8 weeks, cerulein-injected KC and KNeC mice showed increased pancreatic weight compared with the rest of the groups, but no significant difference between them was observed (Supplementary Figure S1E). Saline and cerulein-injected WT and NeC mice displayed no histologic anomalies (Figure 1B). Conversely, saline-injected KC and KNeC mice developed ADMs and low-grade PanINs (Figures 1B, C and Supplementary Figure 1F), consistent with prior findings that NEMO deletion does not affect precancerous lesion formation at the age of 7 weeks [8]. Cerulein administration promoted ADM and PanIN formation in KC and KNeC pancreata, with the absence of NEMO not impacting lesion formation at 8 weeks (Figures 1B and C).

We confirmed that lesions from KNeC mice lacked *Ikbkg* transcription, while cells of the microenvironment, including immune cells and activated stellate cells/fibroblasts, retained *Ikbkg* transcripts (Figure 1A and Supplementary Figure S1G). Cerulein administration did not alter NEMO levels in acinar cells or pancreatic lesions (Supplementary Figure S1H). To evaluate the activation of the NF-κB pathway, we examined the subcellular localization of p65. We detected nucleus-localized p65 in approximately 68.7% of CK19^+^ cells in cerulein-injected KC mice, whereas p65 was virtually cytoplasmic in NEMO-deficient KNeC mice, appearing in their nuclei only in 3.4% of CK19^+^ cells (Supplementary Figure S1I and S1J). Immune cells and stellate cells/fibroblasts exhibited nuclear p65 irrespective of NEMO presence/absence in parenchymal cells (Supplementary Figure S1K).

Considering the role of conventional NF-κB signaling in inflammation, we examined if NEMO deletion affected the immune response via CD45 staining. Cerulein-injected KC mice had more immune cells in the pancreas, while NEMO deletion reduced these numbers (Figure 1D). Quantification of immune sub-populations revealed that cerulein-injected KNeC mice exhibited fewer macrophages and neutrophils compared to KC mice, while no significant difference was observed for B cells and different T cell subtypes (Figure 1E and Supplementary Figure S1L). Next, we examined the localization of immune cells (Supplementary Figure S1M). Macrophages and neutrophils were present in the interlobular and intralobular areas of lesions in both KC and KNeC mice. T cells were detected in both areas in KC mice, whereas they were limited only to the interlobular area in KNeC mice (Figure 1E). B cell localization was inconclusive due to low numbers. In addition, quantitative RT-PCR for F4/80 (*Adgre1*) further confirmed the reduced number of macrophages in KNeC pancreata, while we observed a tendency for higher *Tnf* in KC versus KNeC pancreata (Supplementary Figure S1N). Finally, macrophage polarization (M1/M2) did not differ significantly between KC and KNeC mice (Supplementary Figure S1O).

### NEMO ablation reduces fibrosis and proliferation in precancerous lesions in cerulein-injected 8-week-old KC mice

**Fig. 2:**
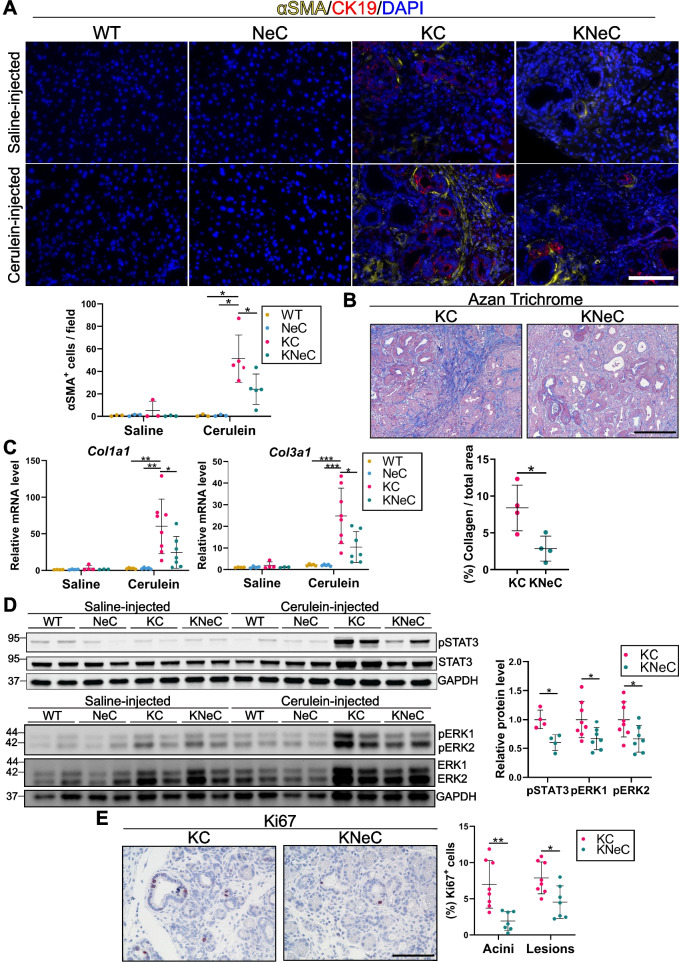
Pancreas-specific NEMO ablation reduces fibrosis in cerulein-injected KC mice at the age of 8 weeks. **A** Top: Visualization of activated stellate cells by αSMA staining on pancreatic sections of saline- or cerulein-injected mice. Scalebar: 100μm. Bottom: Quantification of αSMA^+^ cells. Cerulein-injected KC and KNeC groups: N≥5 mice/group, rest of groups: N≥3 mice/group; t=8 weeks. One-way ANOVA-Tukey for saline-injected groups; One-way ANOVA-Tukey for cerulein-injected groups. **B** Top: Visualization of fibrosis by Azan staining on pancreatic sections of cerulein-injected KC and KNeC mice. Scalebar: 100μm. Bottom: Quantification of area covered by collagen according to Azan staining as a percentage. *N*=4 mice/group; t=8 weeks. Two-tailed Student’s t test. **C** Quantitative RT-PCR for the expression of the indicated transcripts in pancreatic tissue of saline- or cerulein-injected mice, given relative to saline-injected WT mice, which were set to 1. Cerulein-injected KC and KNeC groups: N≥7 mice/group, rest of groups: N≥3 mice/group; t=8 weeks. One-way ANOVA-Tukey for saline-injected groups; One-way ANOVA-Tukey for cerulein-injected groups. **D** Left: Representative pictures of western blots of protein extracts from saline- or cerulein-injected mice. Right: Quantification of western blots for cerulein-injected KC and KNeC mice. Diagrams show the quantification of pERK1/GAPDH, pERK2/GAPDH and pSTAT3/GAPDH ratios, given relative to cerulein-injected KC mice, which were set to 1. N≥4/group; t=8 weeks. Two-tailed Student’s t test. **E** Left: Immunohistochemical analysis of proliferating cells with Ki67 antibody on pancreatic sections of cerulein-injected KC and KNeC mice. Scalebar: 100μm. Right: Percentage of Ki67^+^/total cells of acini and lesions. N≥7 mice/group; t=8 weeks. Two-tailed Student’s t test. Dot plot: Dots represent individual animals. n.s.: *p* > 0.05; *p < 0.05; **p < 0.01; ***p < 0.001

Fibrosis accompanies lesion formation and is linked to PDAC development [24]. To evaluate whether NF-κB is regulating fibrosis, we stained pancreatic sections for αSMA, a marker of activated stellate cells. Pancreata of cerulein-injected KC mice had approximately double the number of αSMA^+^ cells than KNeC mice (Figure 2A and Supplementary Figure S2A). Correspondingly, NEMO deletion led to diminished collagen deposition (AZAN staining) and downregulation of *Col1α1* and *Col3α1* transcription, with a tendency for decreased *Fn1, Tgfb1*, and *Mmp7* levels (Figures 2B, C, and Supplementary Figure S2B).

The IL-6/STAT3 pathway, regulated by NF-κB, is essential for the development of pancreatic lesions and its inhibition reduces lesion numbers [25]. Since NEMO deletion in cerulein-injected KC mice reduced the number of myeloid cells, a source of IL-6, we examined the activation of the IL-6/STAT3 axis [26]. We observed a tendency for reduced *Il6* and a significant decrease in phosphorylated STAT3 in KNeC compared to KC pancreata (Figure 2D and Supplementary Figure S2C). Since this pathway is vital for PanIN development and NEMO deletion appears to impede its activation, PanIN formation may be hindered later in the life of KNeC mice.

Next, we investigated whether the absence of NEMO affected components of the RAS/MAPK pathway, crucial in PDAC establishment [6, 8]. Immunoblotting revealed the presence of oncogenic KRAS in KC and KNeC pancreata regardless of NEMO expression (Supplementary Figure S2D). Importantly, cerulein-injected KC pancreata exhibited highly phosphorylated ERK1 and ERK2, whereas NEMO deletion reduced their activation (Figure 2D and Supplementary Figure S2E). Variability in ERK levels between the same group, particularly in saline-injected KC and KNeC mice, might stem from the stochastic nature of the lesion formation, causing deviations in ADM/PanIN numbers among individual young mice.

Finally, we explored whether these pathway alterations affected cell death or proliferation in ADM and PanIN cells (collectively termed lesion cells) and acinar cells. While no difference in cell death between KC and KNeC lesion cells was observed, Ki67 staining indicated reduced proliferation in acinar and lesion cells in oncogenic KRAS-expressing cells in the absence of NEMO (Figure 2E and Supplementary Figure S2F).

#### NEMO ablation drastically reduces the number of neoplastic lesions in cerulein-injected 10-month-old KC mice

**Fig. 3:**
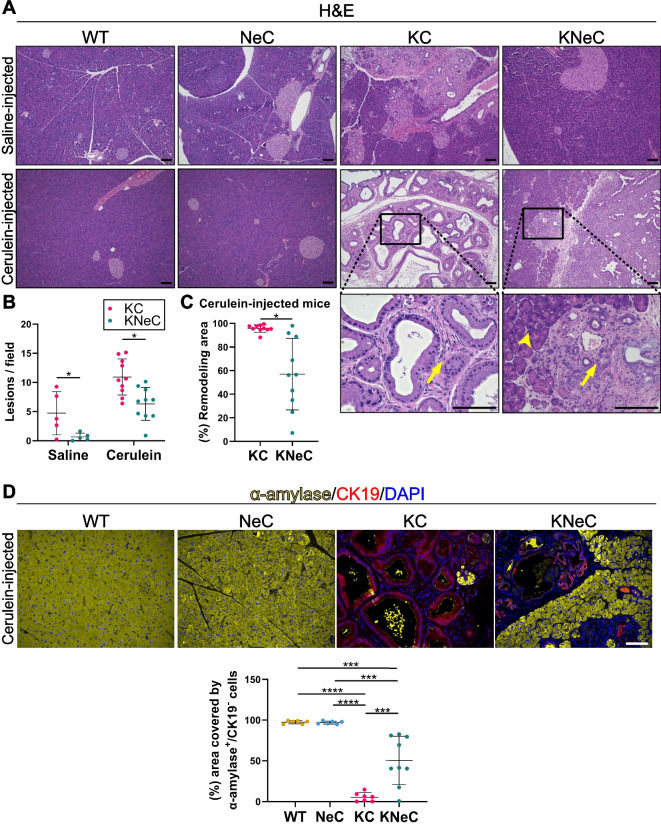
Pancreas-specific NEMO ablation reduces PanIN formation in 10-month-old mice. **A** H&E staining on pancreatic sections of saline- or cerulein-injected mice. Arrow: remodeling area (lesions, desmoplasia); arrowhead: acinar area. Scalebars: 100μm; t=10 months. **B** Quantification of lesions (ADMs, PanINs) on pancreatic sections of saline- or cerulein-injected KC and KNeC mice. Saline-injected groups: N≥5 mice/group, two-tailed Student’s t test; cerulein-injected groups: N≥7 mice/group; t=10 months. Two-tailed Student’s t test. **C** Percentage of remodeling area to total area on pancreatic sections of cerulein-injected KC and KNeC mice. N≥7 mice/group; t=10 months. Two-tailed Student’s t test. **D** Top: Visualization of α-amylase^+^ and CK19^+^ cells on pancreata of cerulein-injected mice. Scalebar: 100μm. Bottom: Percentage of area covered by α-amylase^+^CK19^-^ cells to total cell area. N≥7 mice/group; t=10 months. One-way ANOVA-Tukey. Dot plot: Dots represent individual animals. n.s.: *p* > 0.05; *p < 0.05; **p < 0.01; ***p < 0.001; ****p < 0.0001

Inflammation, fibrosis, proliferation of lesion cells and the STAT3 axis were all downregulated in the absence of NEMO at the age of 8 weeks. Therefore, we hypothesized that the development of PanINs/PDAC might be affected later in the life and analyzed the mice at the age of 10 months. Pancreata from saline-injected KC mice developed multiple PanINs, while absence of NEMO diminished the number (Figure 3A and B). Importantly, the exocrine compartment of pancreata from cerulein-injected KC mice was virtually completely replaced by PanINs, infiltrating immune cells and a strong desmoplastic reaction (Figure 3A). The remodeling area (lesions plus desmoplasia) covered on average 96% of the total field. In contrast, absence of NEMO reduced the remodeling area by 40% and number of lesions by 42%, suggesting that NEMO deletion partially blocked the formation of PanINs (Figure 3B and C). The difference in the percentage of remodeling area was also reflected in their pancreatic weight, with cerulein-injected KC mice displaying a heavier pancreas than KNeC mice (Supplementary Figure S3A).

We next compared the exocrine compartment of KC and KNeC pancreata of cerulein-injected mice by evaluating the presence of amylase, a specific marker of acinar cells. KC pancreata had very few α-amylase^+^/CK19^-^ cells, covering approximately 5% of the total pancreas, while KNeC pancreata still had 50% of the acinar area (Figure 3D). KNeC pancreata also showed increased pancreatic amylase enzymatic activity compared to KC pancreata (Supplementary Figure S3B). These results suggest that NEMO deletion supported the partial preservation of the exocrine compartment and function in the context of oncogenic KRAS expression.

### Pancreas-specific NEMO ablation accelerates the progression of precancerous lesions toward PDAC at 10 months

**Fig. 4:**
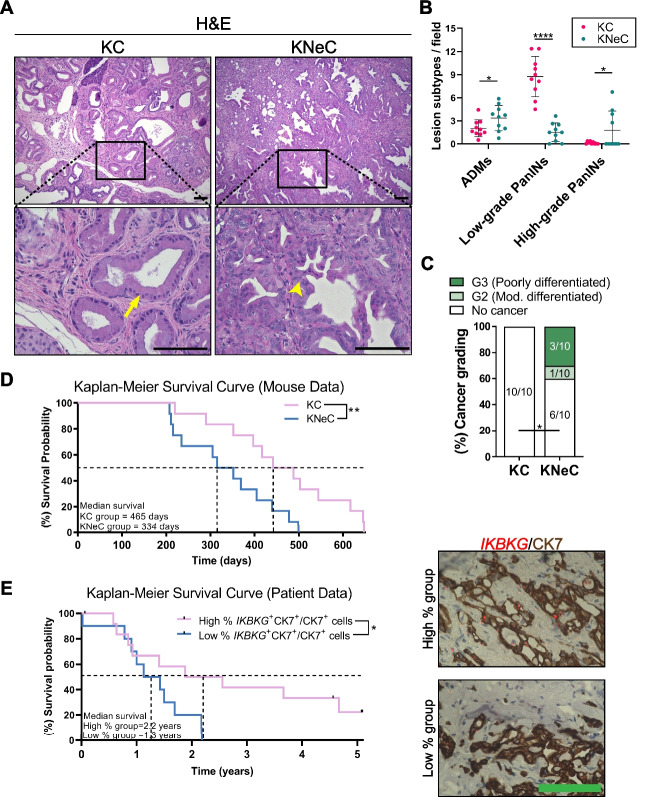
Pancreas-specific NEMO ablation accelerates progression of precancerous lesions to cancer in 10-month-old mice. **A** H&E staining of pancreatic sections from cerulein-injected KC and KNeC mice. Arrow: low-grade PanIN; arrowhead: high-grade PanIN. Scalebars: 100μm; t=10 months. **B** Quantification of ADMs, low-grade and high-grade PanINs on pancreatic sections of cerulein-injected KC and KNeC mice. N≥7 mice/group; t=10 months. Two-tailed Student’s t test. **C** PDAC development and differentiation status in cerulein-injected KC and KNeC mice. A significant difference in tumor incidence was observed between KC and KNeC mice. *N*=10 mice/group; t=10 months. One-tailed Fisher’s exact test. **D** Kaplan-Meier survival curve for cerulein-injected KC (pink line) and KNeC (blue line) mice (*N*=12 mice/group). Log-rank test. **E** Left: Kaplan-Meier survival curve for pancreatic cancer patients comparing group with high percentage of *IKBKG*^+^CK7^+^/ CK7^+^ cells (pink line, *N*=13 patients) to group with low percentage of *IKBKG*^+^CK7^+^/ CK7^+^ cells (blue line, *N*=10 patients). Right: Representative pictures of groups with low- and high-percentage of *IKBKG*^+^CK7^+^/ CK7^+^ cells . Scalebar: 100μm. Log-rank test. Dot plot: Dots represent individual animals. n.s.: *p* > 0.05; *p < 0.05; **p < 0.01; ***p < 0.001; ****p < 0.0001

NEMO deletion preserved the exocrine compartment while reducing the total lesion numbers. Quantification of each lesion subtype revealed that KC pancreata were completely covered by low-grade PanINs and ADMs with a few cases of high-grade PanINs. In contrast, KNeC mice had a six-fold reduction in the number of low-grade PanINs but showed more ADMs. Surprisingly, however, KNeC mice developed significantly more high-grade PanINs than KC mice (Figure 4A and B).

We then examined whether KNeC mice displayed accelerated PDAC development than KC mice. Histological analysis indicated no PDAC in KC mice by the age of 10 months, whereas 40% of KNeC mice already exhibited PDAC (Figure 4C). Investigating tumor differentiation, 10% of 10-month-old KNeC mice had moderately differentiated (G2) PDAC, while 30% displayed poorly differentiated (G3) PDAC (Figure 4C and Supplementary Figure S4A). This accelerated PDAC progression was corroborated by survival analysis. Kaplan-Meier plots revealed that cerulein-injected KC mice had a median survival of 465 days, whereas NEMO ablation shortened their lifespan (median survival=334 days) (Figure 4D).

To assess whether differential NEMO expression also influences human PDAC patient survival, we employed HPA database data. Patients were categorized by survival and *IKBKG* (NEMO) expression level (high or low) from bulk tumors. As in mice, high-*IKBKG* expression in pancreatic cancer patients correlated with longer survival, with a 37% 5-year survival for high-expression cases versus 23% for low-expression cases (Supplementary Figure S4B). Additionally, we explored survival in relation to NEMO expression specifically in neoplastic cells by staining PDAC patient tissue sections for *IKBKG* (NEMO) RNA and CK7, a neoplastic cell marker. Patients with lower percentage of neoplastic cells expressing *IKBKG* (median survival = 1.3 years) showed significantly shorter survival than those with higher percentage of neoplastic cells expressing *IKBKG* (median survival = 2.2 years) (Figure 4E).

In summary, NEMO deletion partially hindered precancerous lesion formation in KC mice. Strikingly, however, its absence promoted progression to high-grade PanINs and PDAC, and was associated with reduced survival, similar to PDAC patients. Our findings suggest a dual role of NEMO in PanIN formation and progression.

### NEMO deletion blocks oncogene-induced senescence in pancreatic lesion cells

**Fig. 5:**
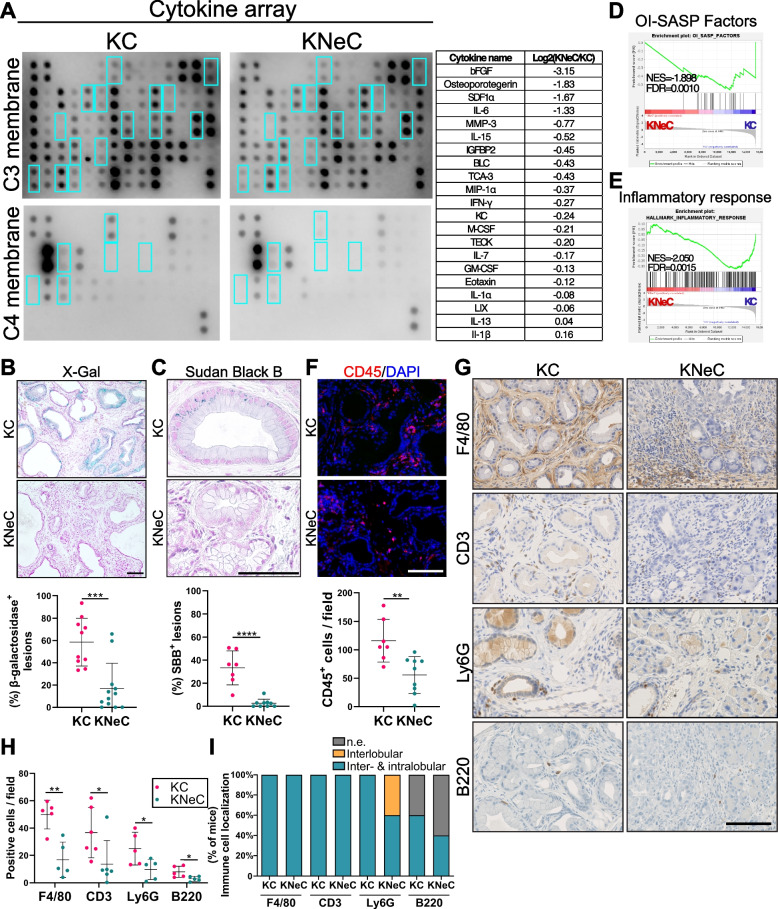
Pancreas-specific NEMO ablation accelerates progression to cancer by blocking oncogene-induced senescence (OIS) in 10-month-old mice. **A** Left: Cytokine array of pancreata from cerulein-injected 10-month-old KC and KNeC mice. OIS-associated cytokines are enclosed in blue borders. Right: Log2 fold change in the expression of OIS-associated cytokines between KC and KNeC pancreata. **B** Top: X-gal staining on pancreas of cerulein-injected KC and KNeC mice. Scalebar: 100μm; t=10 months. Bottom: Percentage of beta-galactosidase^+^ lesions to total lesions. N≥10 mice/group; t=10 months. Two-tailed Student’s t test. **C** Top: Histochemical analysis of senescent cells by Sudan Black B (SBB) on pancreatic tissue of cerulein-injected KC and KNeC mice. Scalebar: 100μm; t=10 months. Bottom: Percentage of SBB^+^ lesions to total lesions. N≥7 mice/group; t=10 months. Two-tailed Student’s t test. **D** GSEA of cerulein-injected KC and KNeC mice for oncogene induced-senescence-associated secretory phenotype (OI-SASP) factors, *N*=3 mice/group; t=10 months. **E** GSEA of cerulein-injected KC and KNeC mice for inflammatory response-associated geneset. *N*=3 mice/group; t=10 months. **F** Top: Visualization of immune cells by CD45 staining on pancreatic sections of 10-month-old cerulein-injected KC and KNeC mice. Scalebar: 100μm. Bottom: Quantification of CD45^+^ cells. N≥7 mice/group. Two-tailed Student’s t test. **G** Visualization of immune cell sub-populations on pancreatic sections of cerulein-injected 10-month-old KC and KNeC mice with antibodies against F4/80, Ly6G, B220 and CD3. Scalebar: 100μm; t=10 months. **H** Quantification of the indicated immune sub-population cells per capture field. *N*=5 mice/group; t=10 months. Two-tailed Student’s t test. **I** Localization of the indicated immune sub-populations. *N*=5 mice/group; t=10 months, n.e. = not examined. Two-tailed Fisher exact test. Dot plot: Dots represent individual animals. n.s.: *p* > 0.05; *p < 0.05; **p < 0.01; ***p < 0.001; ****p < 0.0001

To unravel the mechanism driving the progression of low-grade lesions to PDAC in KNeC mice, we assessed cytokine expression using a cytokine array. Intriguingly, cytokines associated with oncogene-induced senescence (OIS) were diminished in KNeC pancreata compared to KC pancreata (Figure 5A and Supplementary Table S3). OIS serves as a safeguard mechanism that attenuates the progression of precancerous lesions towards cancer. It can be induced by oncogenic KRAS expression at low-grade PanINs and block their progression to high-grade PanINs/PDAC [27]. We examined senescence-associated β-galactosidase (SA-β-Gal) activity, a commonly used marker of senescence, by X-Gal staining. In KC mice, the majority of PanINs were scored as positive (approximately 59%), while this number was drastically reduced in the absence of NEMO (17% SA-β-Gal^+^ lesions) (Figure 5B). In addition, pancreatic sections were stained with Sudan Black B (SBB) to detect lipofuscin, a product of oxidized biomolecules accumulating in senescent cells [28]. Similarly, 33% of KC lesions were stained by SBB, while only 3% of KNeC lesions were positive (Figure 5C).

To address whether the difference in PanIN numbers and their senescent phenotype is already present at a younger age, we analyzed the mice at the age of 6 months. As expected, we observed that the remodeling area was higher in KC than in KNeC pancreata while we did not observe any difference in cell death of lesion cells (Supplementary Figures S5A, S5B and S5C). Further, KC mice developed more PanINs, but KNeC mice were more prone to develop higher-grade PanINs already at the age of 6 months (Supplementary Figure S5D and Supplementary Figure S5E). Finally, X-Gal staining revealed the presence of senescence in 47% of KC PanINs, while only 18% of KNeC PanINs were marked as positive (Supplementary Figure S5F). These results were further verified by examining the transcription of senescence-, inflammation- and fibrosis-associated genes (Supplementary Figure S5G). The increase in the percentage of X-gal^+^ PanINs in KC mice indicated that senescent PanINs accumulated from 6 to 10 months (+12%). During the same period, an only slight reduction (-1%) was observed in KNeC mice. This suggests that the absence of NEMO blocks senescence in PanIN lesions.

Next, we investigated the regulation of OIS in the absence of NEMO in 10-month-old mice by comparing the expression profile of KC and KNeC lesions. We used laser capture microdissection to isolate pancreatic lesions from 10-month-old mice and performed RNAseq [29]. Notably, a signature associated with the PDAC development (Neoplasm_of_the_pancreas) was highly enriched in KNeC compared to KC lesions (NES=1.828) (Supplementary Figure S5H), indicating a more advanced neoplastic feature.

Senescence-associated secretory phenotype (SASP) genes, often upregulated in senescent cells, contribute to an inflammatory microenvironment through positive feedback. To assess the impact of NEMO ablation on SASP gene transcription, a geneset comprising 27 SASP genes that are upregulated during OIS was examined [21]. Interestingly, NEMO deletion depleted this signature (NES=–1.898), highlighting that the absence of NEMO disrupts the positive feedback loop between SASPs and senescence (Figure 5D).

Since NEMO deletion reduced SASP transcription, we questioned whether inflammation was also affected. First, the hallmark-GSEA signature of the inflammatory response was significantly reduced in KNeC compared to KC lesions (NES=–2.05) (Figure 5E). Additionally, CD45 staining demonstrated immune cell infiltration in KC pancreata, while NEMO-deficient mice exhibited a weaker immune reaction (Figure 5F). Characterization of the immune cell sub-populations revealed in both KC and KNeC pancreata macrophages (F4/80^+^) as the most prominent population, followed by T (CD3^+^) cells, neutrophils (Ly6G^+^ cells), and B (B220^+^) cells (Figure 5G and H). Quantification of T cell subtypes revealed reduced CD4^+^ cells and a strong tendency for reduced CD8^+^ cells in the absence of NEMO while no difference was observed for FoxP3^+^CD45^+^ cells (Supplementary Figure S5I). Further, we compared the transcription of Th1-, Th2- and Th17-assosiated genes. In general, we observed a tendency of reduced transcription of these genes in the absence of NEMO, but with high variability (Supplementary Figure S5J). While all immune sub-populations were detected in interlobular and intralobular areas of neoplastic lesions in KC and KNeC mice, some KNeC mice lacked intralobular neutrophil infiltration (Figure 5I). Interestingly, we observed that Ly6G^+^ cells were more attracted toward ADM/duct-like lesions than PanINs (Supplementary Figure S5K). It is possible that specific secretion of cytokines/chemokines is altered when cells enter the PanIN stage, reducing the attraction to neutrophils. Further, immune cells were not observed at the core of tumors in KNeC mice with cancer development, likely due to desmoplasia. Finally, the polarization of macrophages did not significantly differ between KC and KNeC mice (Supplementary Figure S5L).

Oncogene-induced senescence is associated with upregulation of the tricarboxylic acid cycle (TCA), oxidative phosphorylation and lipid metabolism. These changes are essential for stable senescence-associated cell growth arrest, and overcoming these shifts could lead to tumorigenesis [30]. Using our RNAseq results, we identified that genesets associated with TCA, oxidative phosphorylation and lipid metabolism were depleted in the absence of NEMO, likely due to the higher grade of PanINs in KNeC mice (Supplementary Figure S5J).

### NEMO ablation accelerates proliferation and increases DNA damage in pancreatic lesion cells

**Fig. 6:**
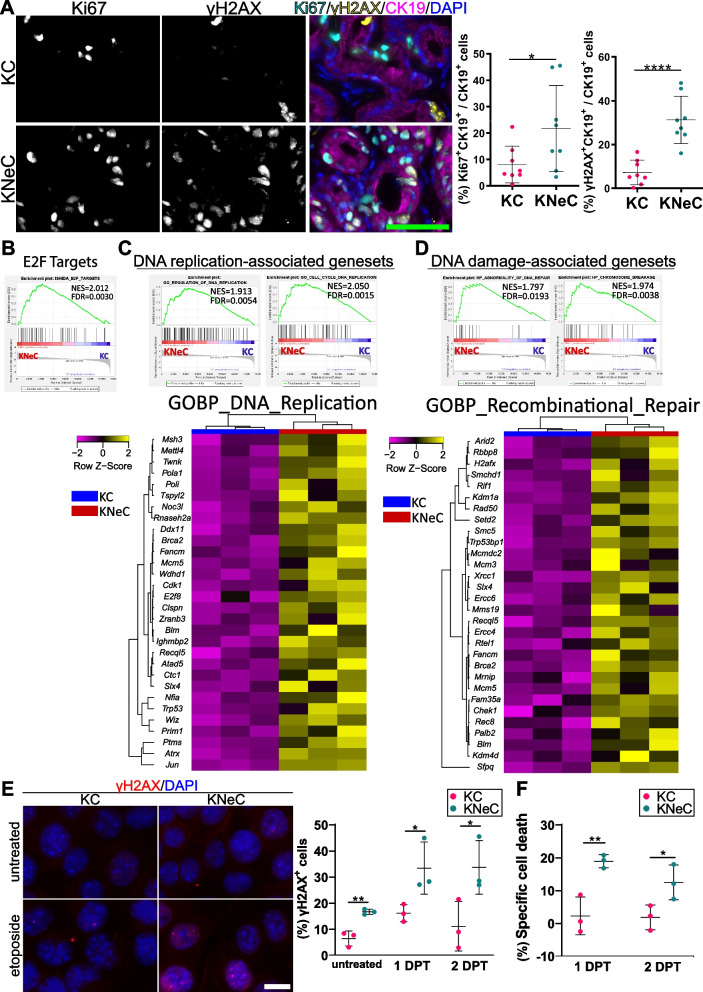
NEMO ablation increases proliferation and DNA damage in lesion cells of 10-month-old mice. **A** Left: Visualization of Ki67^+^, γH2AX^+^ and CK19^+^ cells on pancreatic sections of 10-month-old cerulein-injected KC and KNeC mice. Scalebar:50μm. Right: Percentage of Ki67^+^CK19^+^ cells and γH2AX^+^CK19^+^ cells to total CK19^+^ cell number. *N*=8 mice/group; t=10 months. Two-tailed Student’s t test. **B** GSEA of cerulein-injected KC and KNeC mice for the E2F target-associated geneset. *N*=3 mice/group; t=10 months. **C** Heatmaps and GSEA of cerulein-injected KC and KNeC mice for DNA replication-associated genesets. *N*=3 mice/group; t=10 months. **D** Heatmaps and GSEA of cerulein-injected KC and KNeC mice for DNA damage-associated genesets. *N*=3 mice/group; t=10 months. **E** Left: Visualization of γH2AX^+^ cells on untreated and etoposide-treated KC and KNeC primary cancer cells. The etoposide-treated cells were visualized 2 days post treatment. Scalebar: 10μm. Right: Quantification of untreated or etoposide-treated γH2AX^+^ cells analyzed 1- or 2-days post treatment, *N*=3 independent experiments/primary cell culture. Two-tailed Student’s t test. **F** Quantification of specific cell death due to etoposide 1- and 2-days after treatment, *N*=3 independent experiments/primary cell culture. Two-tailed Student’s t test. Dot plot: **A** Dots represent individual animals, (**E**, **F**) Dots represent independent experiments. n.s.: *p* > 0.05; *p < 0.05; **p < 0.01; ***p < 0.001; ****p < 0.0001

Despite reduced lesion numbers and inflammation, KNeC mice exhibited accelerated PDAC development and poorer survival. Since NEMO deletion blocked senescence in lesions, we investigated their proliferation rate and observed a higher number of Ki67^+^ lesion cells in the absence of NEMO (Figure 6A). In line, our RNAseq results indicated the enrichment of DNA replication-associated and E2F-regulated genes in the absence of NEMO (Figure 6B, C and Supplementary Figure S6A).

DNA damage and, subsequently, accumulation of mutations, are essential for the progression of low-grade toward high-grade PanINs and are supported by increased proliferation [31]. Staining against γH2AX, a DNA damage marker, revealed an increased level of DNA damage in lesions in the absence of NEMO, indicating an impaired response against DNA damage (Figure 6A). Further, genesets associated with recombinational repair, abnormality of DNA repair and chromosomal breakage were highly enriched in the absence of NEMO (Figure 6D and Supplementary Figure S6B).

We next investigated if p53 activities were altered in PanIN lesions with high DNA damage. First, we quantified the total number of PanIN lesion cells with nuclear p53, observing 7.3% p53^+^ cells in KC lesions and 39.1% in KNeC lesions (Supplementary Figure S6C). These results align with the reports that high-grade lesions tend to have increased p53 levels [32, 33]. Notably, p53^+^ KC lesion cells exhibited a weak signal, while KNeC lesion cells showed variability in signal strength. We then assessed nuclear p53 signal in γH2AX^+^ lesion cells, detecting it in 40.5% of KC and 67.5% of KNeC cells (Supplementary Figure S6D). In both KC and KNeC mice, nuclear p53 was enriched in γH2AX^+^ cells. The higher frequency of nuclear p53 in γH2AX^+^ cells in the KNeC samples may again reflect stronger DNA damage in KNeC PanINs. Finally, we analyzed if the presence of p53 was crucial in OIS. We performed staining on serial sections with p53 and SBB. We detected SBB^+^ cells with and without p53 presence, indicating that p53 was not a reliable marker for senescent PanIN cells (Supplementary Figure S6E).

These results highlight that NEMO ablation promoted DNA damage accumulation in precancerous lesion cells. Considering the distinct mutational backgrounds between precancerous lesions and PDAC, we explored NEMO's role in DNA damage accumulation in cancer cells specifically. We used primary cancer cells from KC and KNeC mice and assessed DNA damage under normal and etoposide-treated conditions. γH2AX staining revealed that NEMO deletion supported the accumulation of γH2AX^+^ foci as a result of accumulated DNA damage under non-treated conditions. The accumulation of DNA damage in both KC and KNeC primary cancer cells was further increased one- and two-days post etoposide treatment, preserving its significant difference between KC and KNeC cells (Figure 6E).

These findings indicate impaired DNA damage response (DDR) and repair in neoplastic KNeC cells. Next, we evaluated whether DDR deficiency due to NEMO deletion sensitized KNeC cells to etoposide, potentially inducing synthetic lethality. FACS analysis with the cell death markers propidium iodide (PI) and Annexin V revealed that etoposide treatment significantly elevated specific cell death in NEMO-deficient cells 1- and 2-days post-treatment (DPT) (Figure 6F). Therefore, the combination of NEMO deletion and etoposide treatment promoted cell death, suggesting a beneficial effect in killing pancreatic cancer cells.

## Discussion

The essential role of the conventional NF-κB pathway in cancer development is widely acknowledged [34]. NF-κB governs pro-proliferative, anti-apoptotic, and inflammation-regulating genes, rendering it a promising target for cancer therapy. However, despite its prevalent oncogenic functions, the role of NF-κB is context-dependent [35]. For instance, in pancreatic cancer, the conventional NF-κB pathway is active and suggested to support cancer development [5, 6, 36, 37]. Interestingly, however, analysis of human patient data reveals that decreased NEMO expression correlates with unfavorable prognosis [23]. Therefore, the role of NF-κB has to be examined specifically in each scenario.

Our study demonstrates that while NEMO is not required for the initial lesion development in KC mice, it does contribute to immune and fibrotic responses that regulate PanIN formation [24]. Examination of 10-month-old mice indicated that KC pancreata exhibited extensive neoplastic coverage, whereas NEMO deficiency strongly reduced the development of neoplastic lesions. This suggests that a prolonged inflammatory/fibrotic microenvironment is pivotal for PanIN formation, manifesting over an extended timeframe. These findings align with prior research on a pancreas-specific IKK2 deletion model which described that NF-κB signaling supports PanIN formation [6].

On the other hand, our study demonstrates that NEMO deletion diminishes PanIN formation while unexpectedly supporting PanIN progression via blocking OIS. Notably, oncogenic KRAS-driven PanINs in KC mice typically enter OIS, stopping their progression. These OIS cells release SASPs, fostering immune cell infiltration and low-grade PanIN formation in a positive feedback loop. In the absence of NEMO, reduced senescent PanINs can result in diminished SASP secretion, driving proliferation and DNA damage, thus accelerating PDAC development. Overall, NEMO exhibits dual roles, both oncogenic and tumor-suppressive, in 10-month-old KC mice. Our findings challenge the classical view that stronger inflammation and higher levels of precancerous lesions are associated with a higher chance of PDAC development.

Of note, the dilemma of “blocking or escaping” PanINs from senescence in the absence of NEMO is not entirely clear. Interestingly, the percentage of senescent PanINs in KNeC mice at 6 and 10 months is similar, whereas the KC group experiences a notable increase during the same interval. This suggests potential senescent PanIN accumulation in the KC group and a continuous “escape” due to NEMO deletion. However, validation of the constant “escape” hypothesis requires evidence showcasing the accumulation of proliferating cells that carry a ‘stamp’ of senescence.

Our results align with prior studies while also revealing discrepancies. Previously, it has been demonstrated that IKK2 deletion not only reduces PanINs by 25% but also curtails PDAC development by 60% [10]. Conversely, NEMO deletion decreased PanINs by 42%, yet paradoxically elevated the susceptibility to PDAC development. These differences could stem from the degree of NF-κB inhibition following NEMO versus IKK2 deletion. Previous studies show that NEMO knockout almost entirely abolishes TNFα-induced NF-κB activity, unlike IKK2 knockout, where substantial activity was retained [16, 38]. Thus, PDAC dynamics post NEMO vs. IKK2 deletion might differ. Both knockouts lower PanIN numbers, but the stronger effect of NEMO deletion results from more robust NF-κB pathway inhibition. A weaker NF-κB inhibition by IKK2 knockout could allow residual active NF-κΒ signaling that triggers OIS. In contrast, NEMO knockout robustly suppresses the NF-κB pathway, eliminating the OIS barrier and accelerating PDAC progression. A similar observation was made in hepatocellular carcinoma where NEMO deletion in hepatocytes completely blocked NF-κB activation resulting in increased liver tumorigenesis, while such an effect was not observed in IKK2 deletion [39]. There is also increasing evidence showing that NEMO can demonstrate an NF-κB independent function [40, 41]. In these studies, an absence of NEMO was shown to regulate cell death in hepatocytes and intestinal epithelium through an interaction with RIPK1. The possibility of an NF-κB independent function of NEMO in PDAC still requires further elucidation, since increased cell death through apoptosis was not observed in KNeC mice.

In another study, RelA knockout blocks OIS in PanINs and their SASP expression while promoting additional low-grade PanIN formation [11]. While the inhibition of OIS from RelA knockout is comprehensible, other outcomes such as the increased formation of low-grade PanINs in parallel to SASP reduction are more difficult to be reconciled.

Finally, NEMO deletion supported DNA damage accumulation not only in precancerous lesions but also directly in cancer cells. Intriguingly, etoposide treatment moderately only increased KC cancer cell death but significantly raised KNeC cancer cell death. We hypothesize that since the NF-κB pathway, a DDR regulator, is suppressed in KNeC cells, etoposide efficacy increases similarly to synthetic lethality.

## Conclusions

In summary, our study provides evidence underscoring the context-dependent dual role of conventional NF-κB signaling during PDAC development, which displays both oncogenic and tumor-suppressive properties. The broad effectiveness of anti-NF-κB agents may render PDAC treatment ineffective; however, a targeted therapeutic approach directed at high-grade lesions and PDAC cells, when coupled with DNA damage-inducing agents, holds potential for improving patient lifespan.

## Supplementary Information

**Additional file 1.** GSEA genesets from GO_BP, GO_MF and KEGG databases enriched in KC vs KNeC lesions and KNeC vs KC lesions.

**Additional file 2.**

**Additional file 3.**

## Data Availability

The RNAseq data supporting the conclusions of this article is available in the following repository: ArrayExpress repository (E-MTAB-12433), link: https://www.ebi.ac.uk/biostudies/studies/E-MTAB-12433?key=497771df-956c-468c-b97e-8ea773a9b632.

## Abbreviations

Adgre1: Adhesion G Protein-Coupled Receptor E1
ADM: Acinar-ductal metaplasia
ANOVA: One-way analysis of variance AP-1
AP1: Activator protein 1
AZAN: Heidenhain’s azocarmine aniline
CK19: Cytokeratin 19
Col1a1: Collagen, type I, alpha 1
Col3a1: Collagen, type III, alpha 1
DDR: DNA damage response
DMEM: Dulbecco's modified eagle medium
DPT: Days post treatment
EMSA: Electrophoretic mobility shift assay
FACS: Fluorescence-activated cell sorting
FBS: Fetal bovine serum
FFPE: Formalin fixed paraffin embedded
Fn1: Fibronectin 1
FPKM: Fragments per kilobase of transcript per million
GAPDH: Glyceraldehyde 3-phosphate dehydrogenase
GSEA: Gene set enrichment analysis
H2AX: H2A histone family member X
H&E: Hematoxylin and eosin
HBSS: Hank’s balanced salt solution
HEP: Humane endpoint
HPA: Human protein atlas
IF: Immunofluorescence
IHC: Immunohistochemistry
IKBKG: Inhibitor of kappa B kinase regulatory subunit gamma
IL-1: Interleukin 1
IL-6: Interleukin 6
IκB-α: Inhibitor of κBα
KC: Pdx1-Cre;KRASG12D
KNeC: Pdx1-Cre;LSL-KRASG12D;NEMOfl/fl
KRAS: Kirsten rat sarcoma virus
LCM: Laser capture microdissection
Ly6G: Lymphocyte antigen 6 complex locus G6D
Mmp7: Matrix metallopeptidase 7
NeC: Pdx1-cre;NEMOfl/fl
NEMO: NF-κB essential modulator
NF-κB: Nuclear factor kappa-light-chain-enhancer of activated B cells
OIS: Oncogene-induced senescence
PanIN: Pancreatic intraepithelial neoplasm
PBS: Phosphate-buffered saline
PDAC: Pancreatic ductal adenocarcinoma
Pdx1: Pancreatic and duodenal homeobox 1
PFA: Paraformaldehyde
PI: Propidium Iodide
qRT-PCR: Quantitative real time polymerase chain reaction
RNAseq: RNA sequencing
RPL13: Ribosomal protein L13
SASP: Senescence-associated secretory phenotype
SA-β-Gal: Senescence-associated β-galactosidase
SBB: Sudan black B
SDS: Sodium dodecyl sulfate
STAT3: Signal transducer and activator of transcription 3
TCA: Tricarboxylic acid cycle
TGFβ: Transforming growth factor β
Tnf: Tumor necrosis factor
WB: Western blot
WT: Wild type
