# Supplementary material for: NEMO/NF-κB signaling functions as a double-edged sword in PanIN formation versus progression to pancreatic cancer
Source: Mol Cancer. 2024 May 16;23:103. doi: 10.1186/s12943-024-01989-x (PMC11097402; doi:10.1186/s12943-024-01989-x)

Figure 2D and S1B

Top to bottom appearance order: **pSTAT3** and **NEMO**

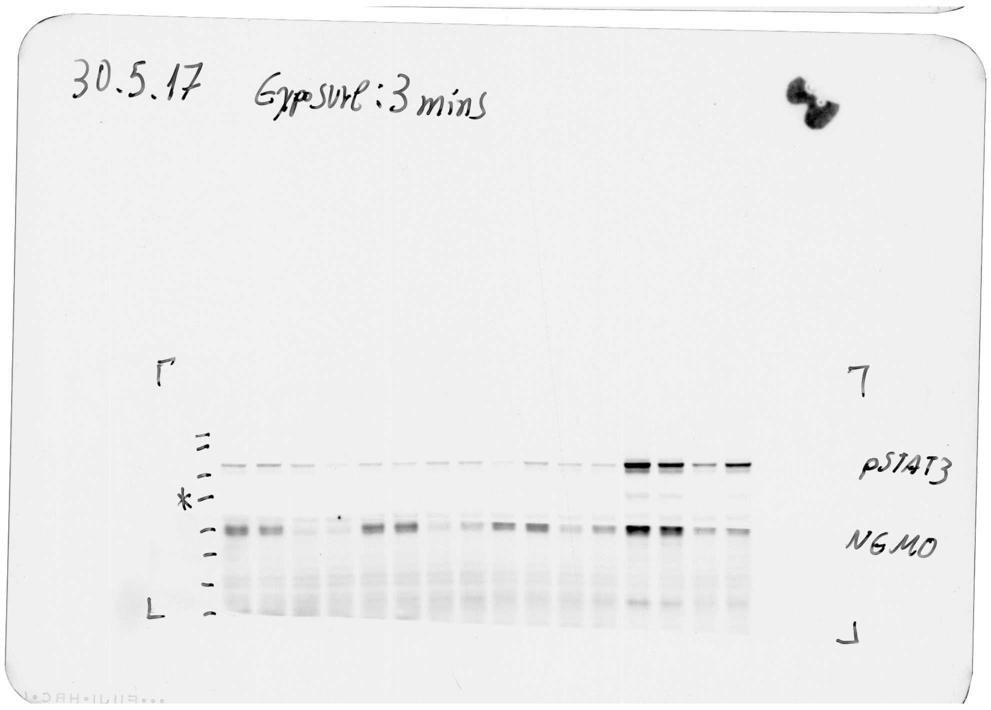

For **STAT3** only

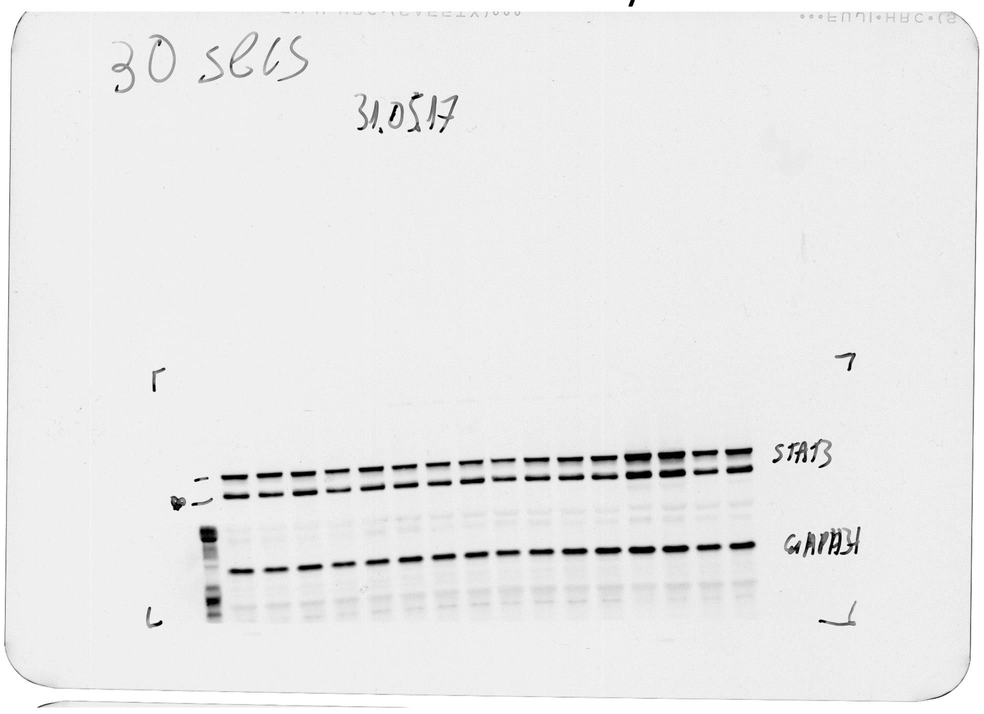

For **GAPDH** only

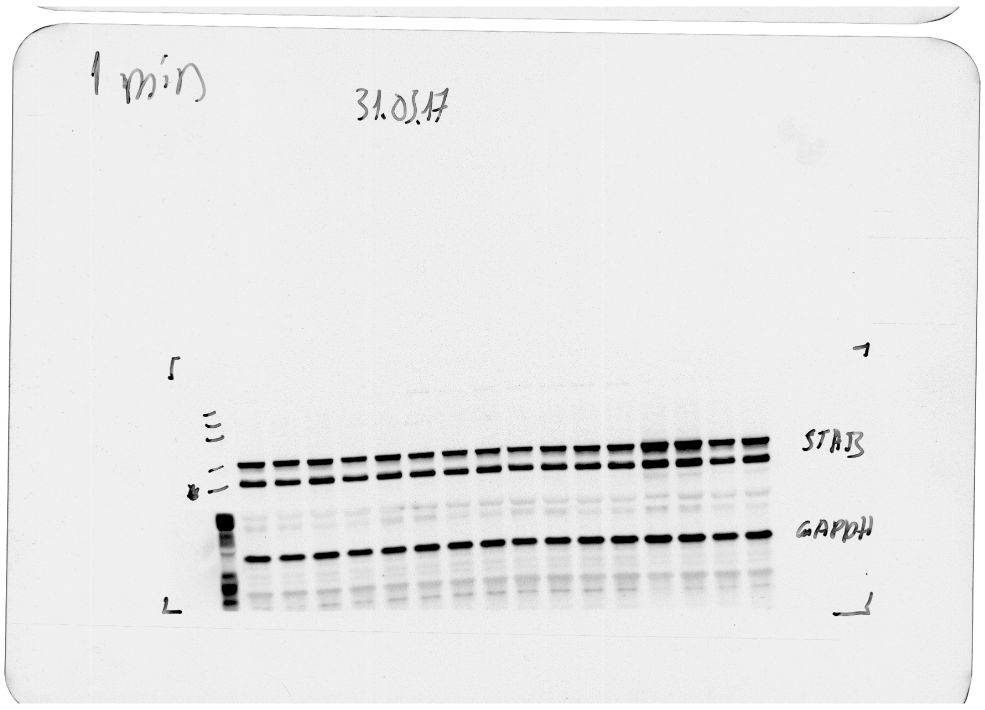

Total Scan (Colorimetric)

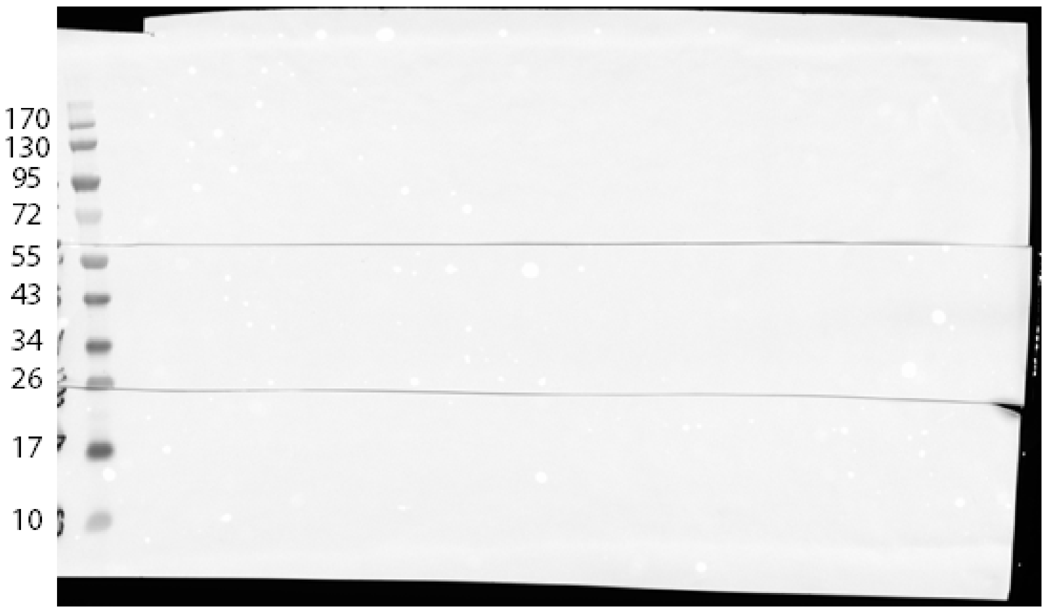

Figure 2D

Top to bottom appearance order: **pERK1** and **pERK2**

Chemiluminescence

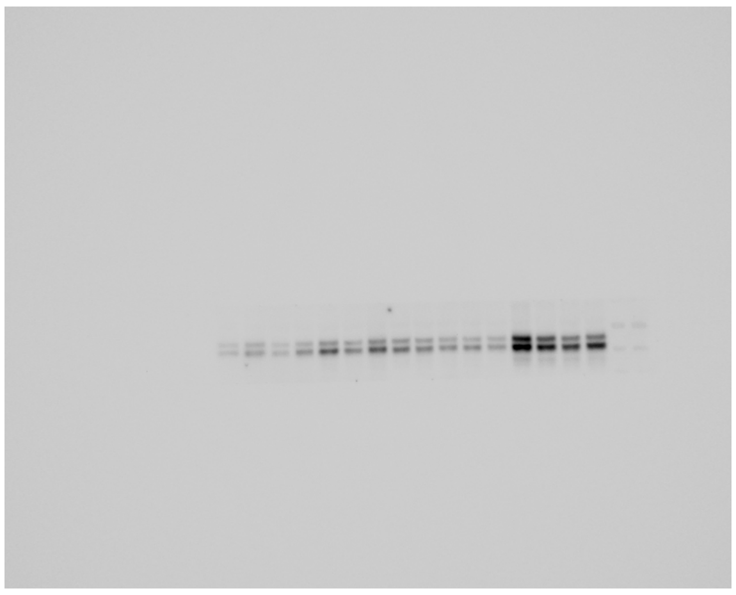

Colorimetric (Scan)

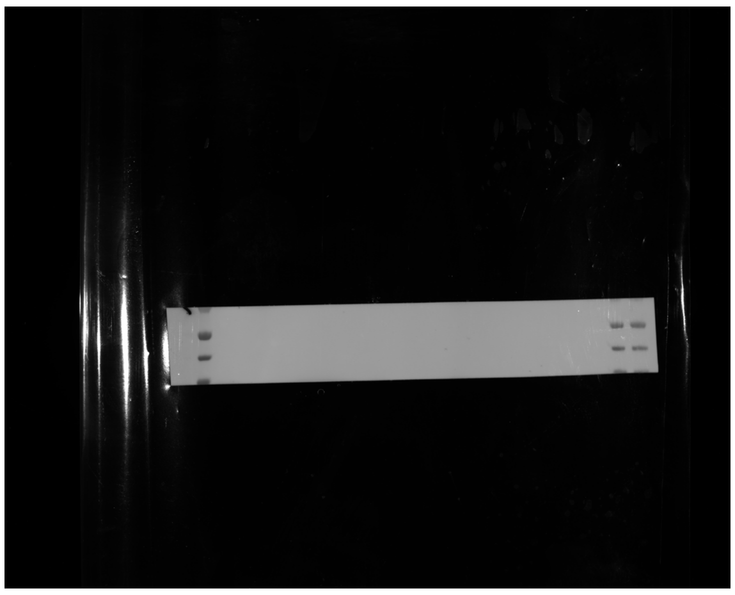

Overlay
(Chemiluminescence plus Scan)

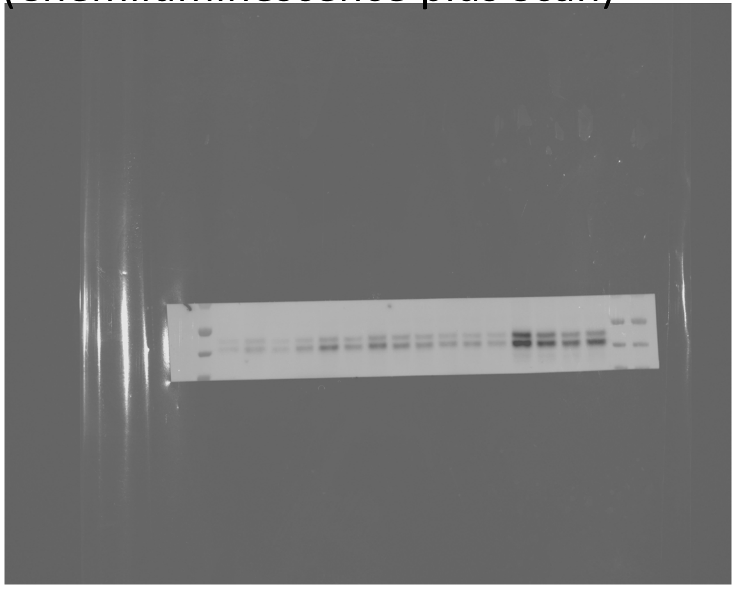

Top to bottom appearance order: **ERK1**, **ERK2** and **GAPDH**

Chemiluminescence

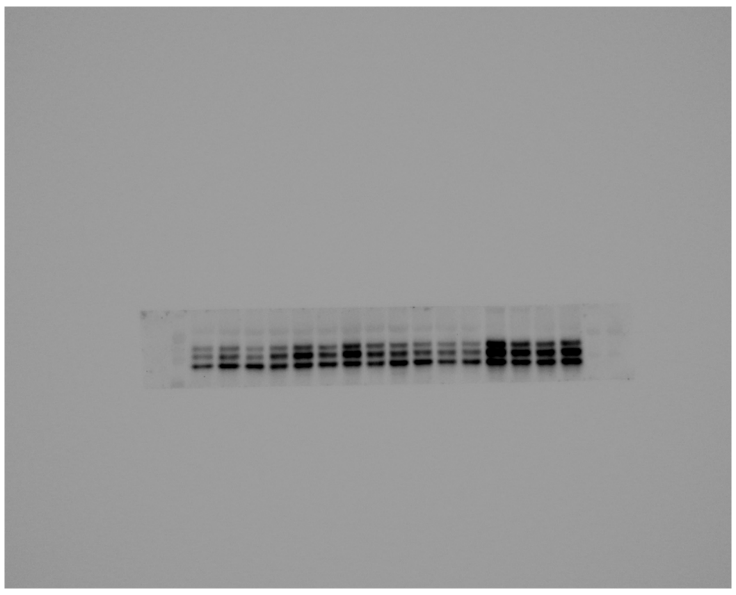

Colorimetric (Scan)

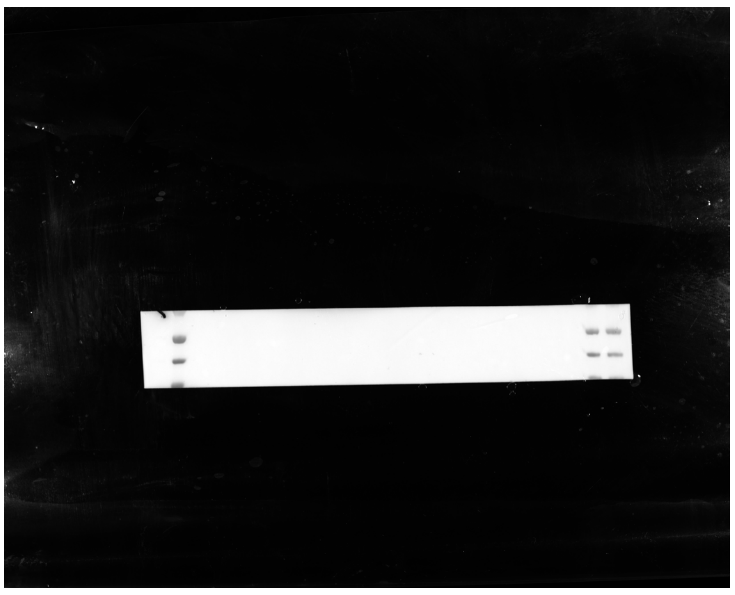

Overlay
(Chemiluminescence plus Scan)

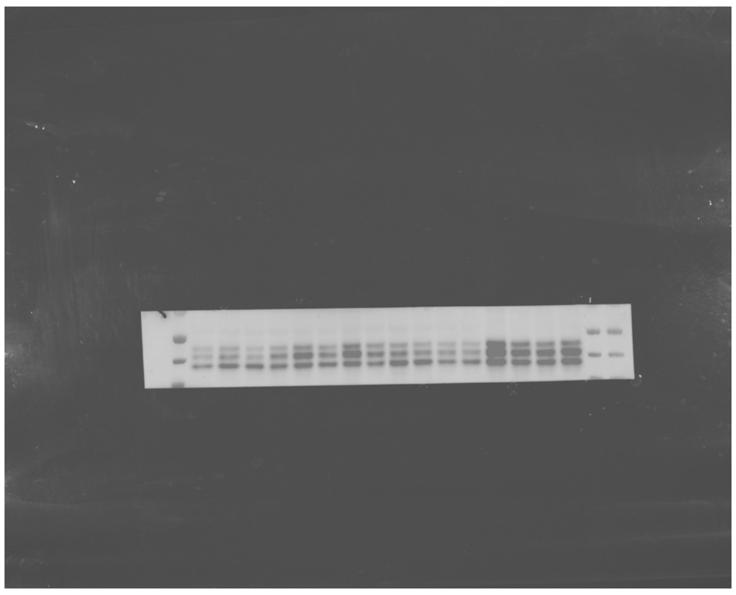

Total Scan (Colorimetric)

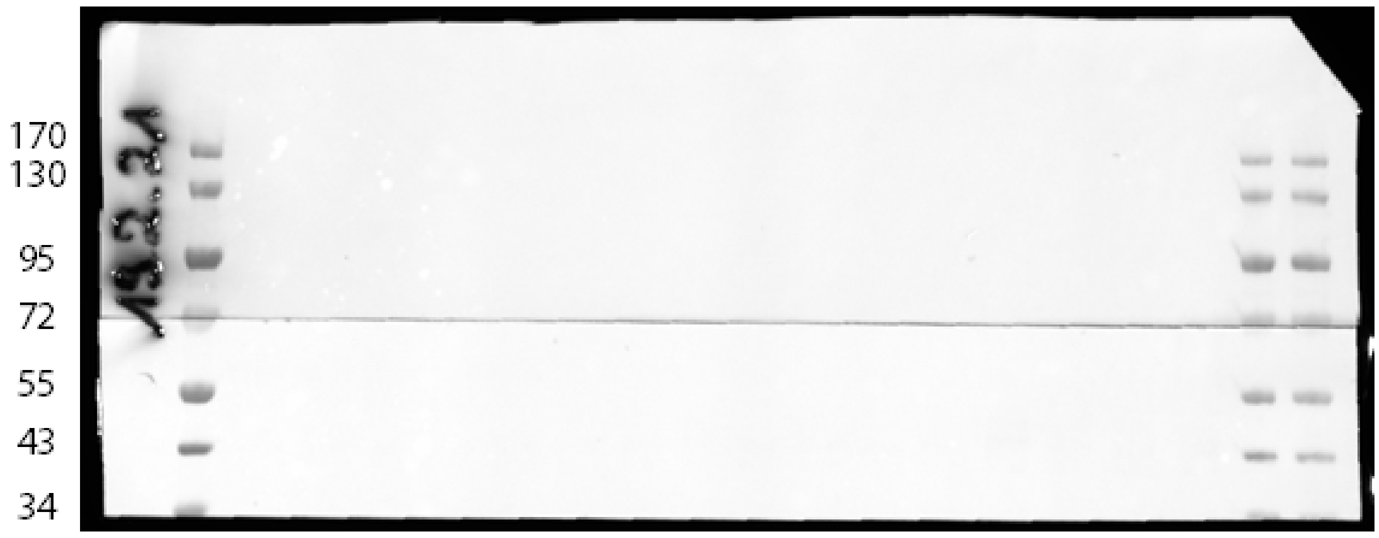

Figure 5A

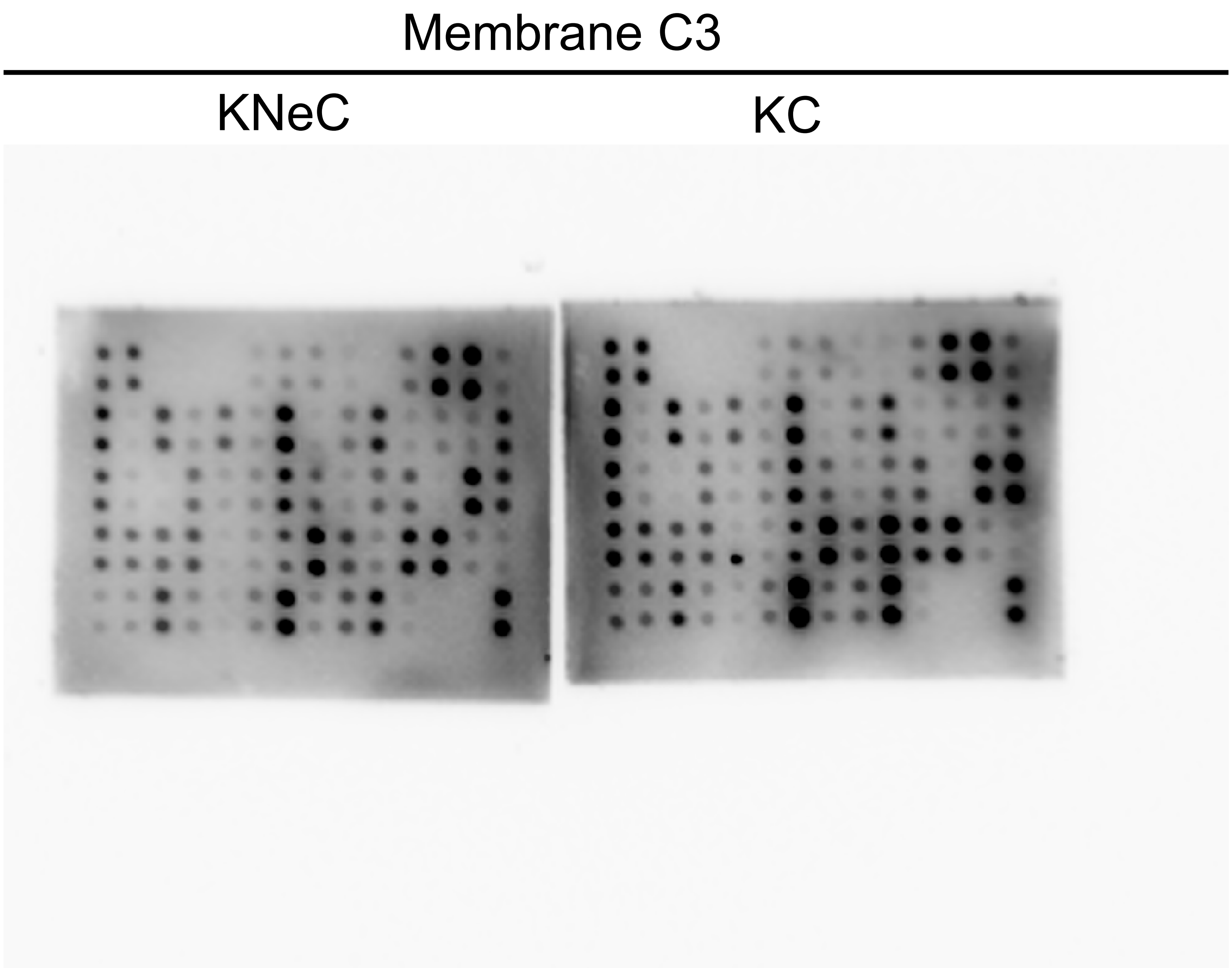

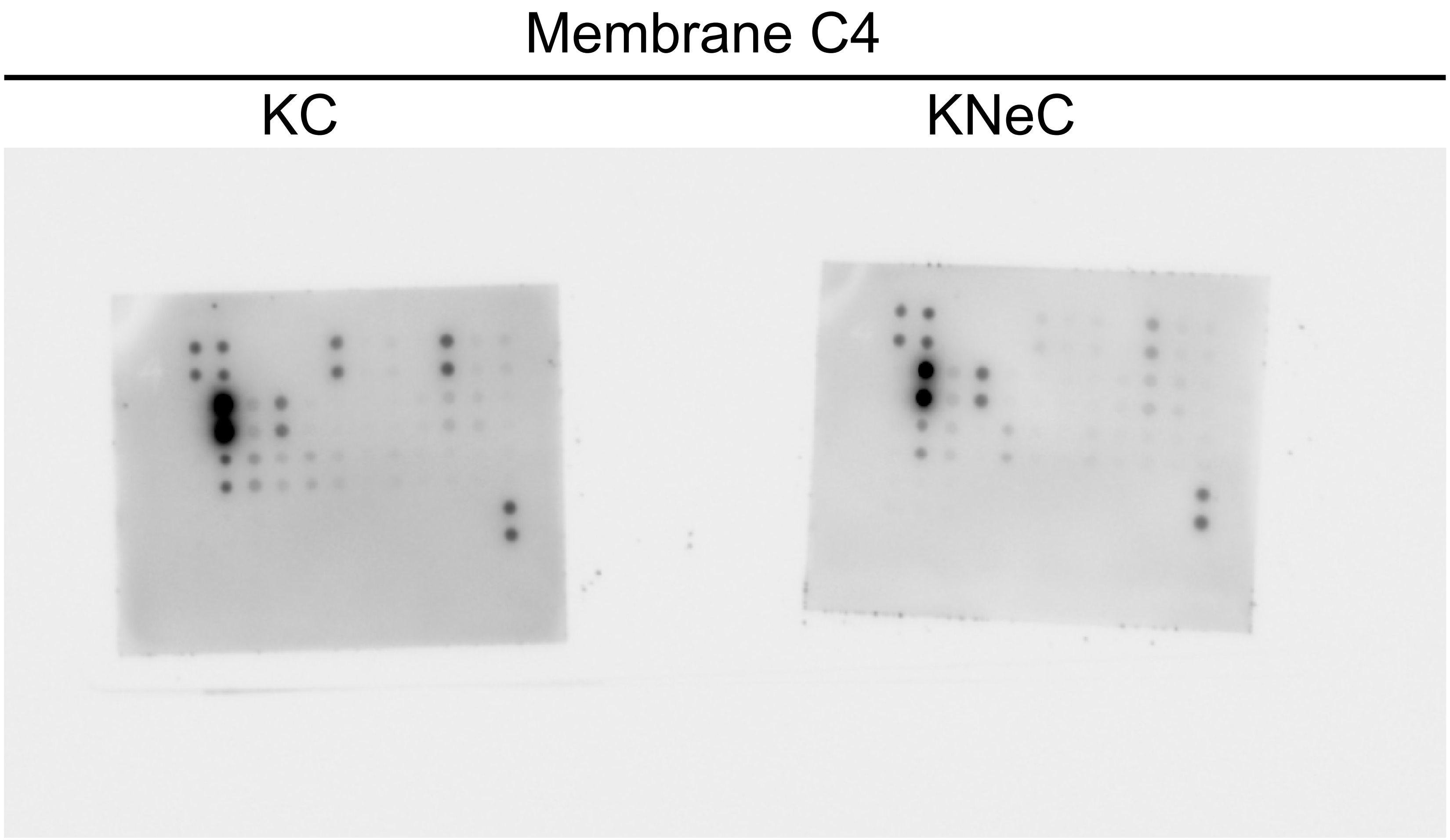

Figure S1D

EMSA

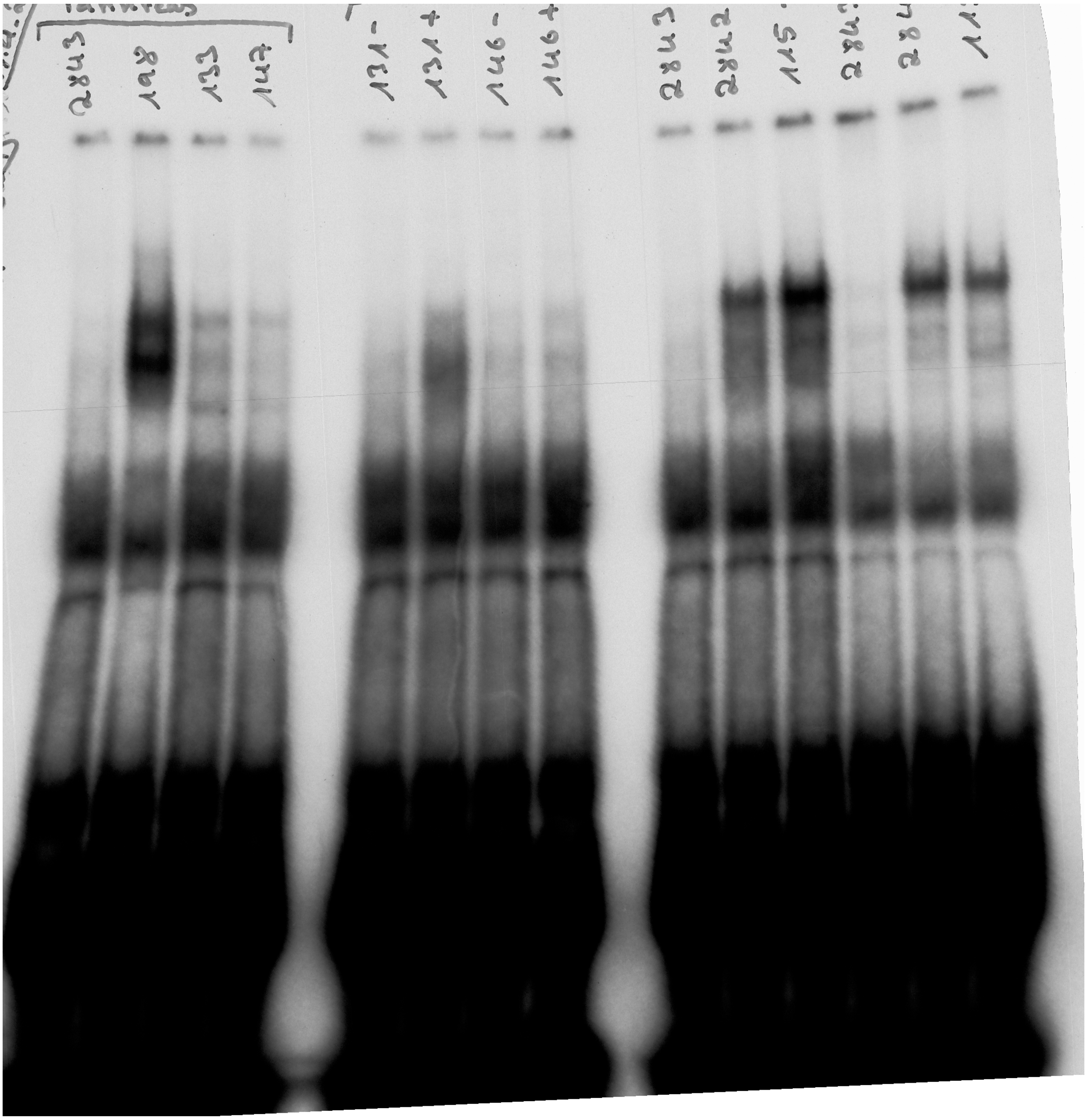

Figure S2D

GAPDH

Chemiluminescence

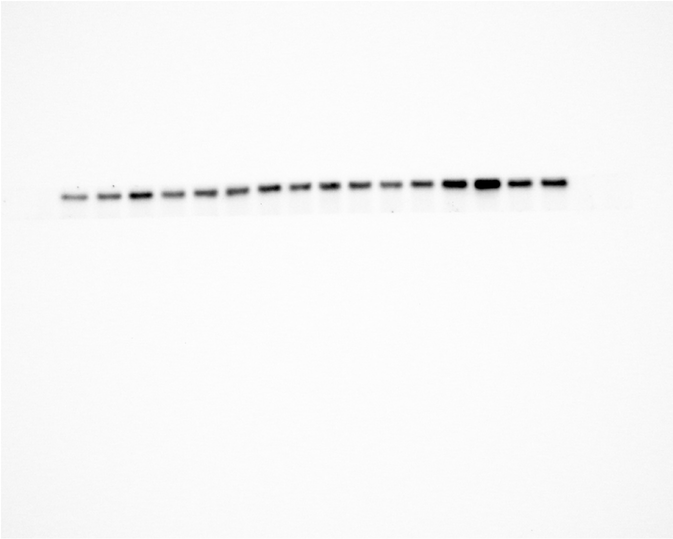

Colorimetric (Scan)

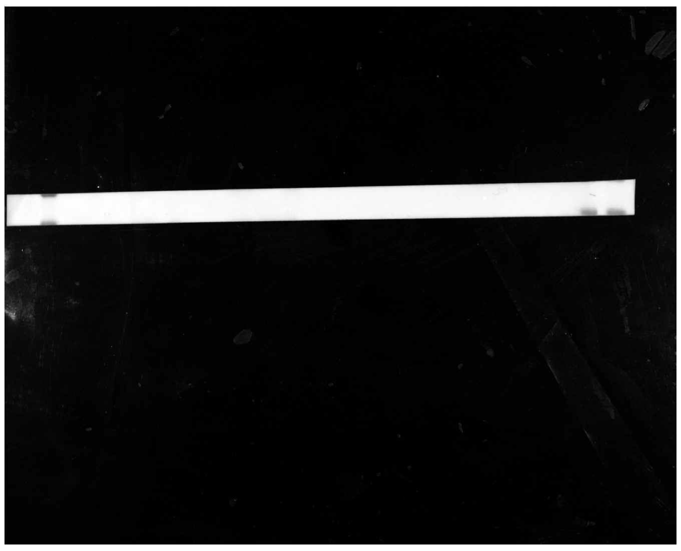

Overlay
(Chemiluminescence plus Scan)

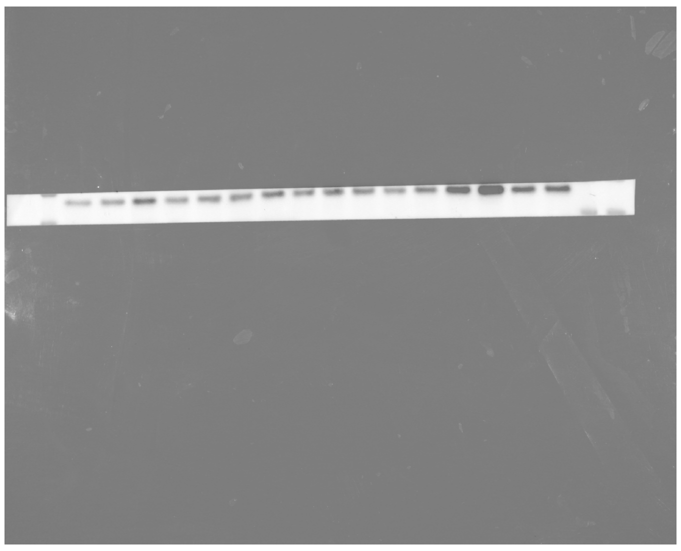

RASG12D

Chemiluminescence

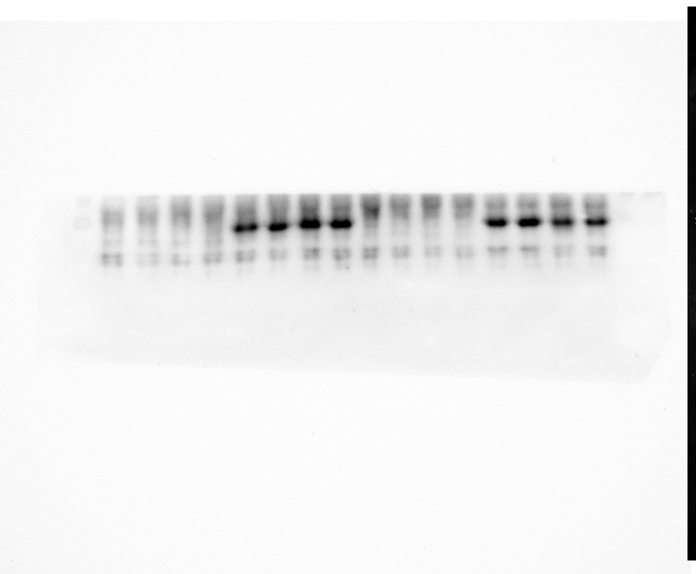

Colorimetric (Scan)

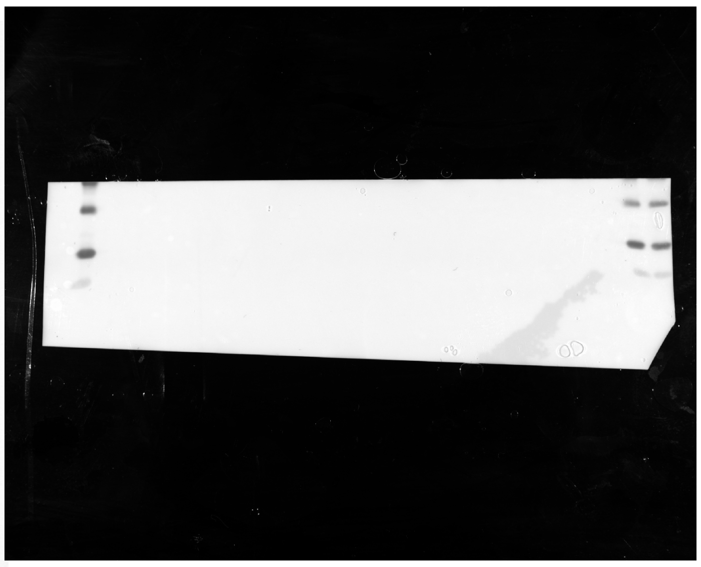

Overlay
(Chemiluminescence plus Scan)

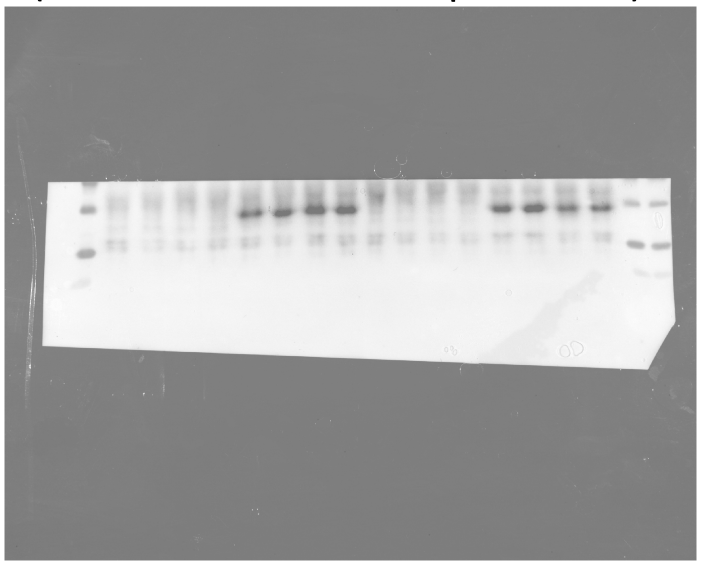

Total Scan (Colorimetric)

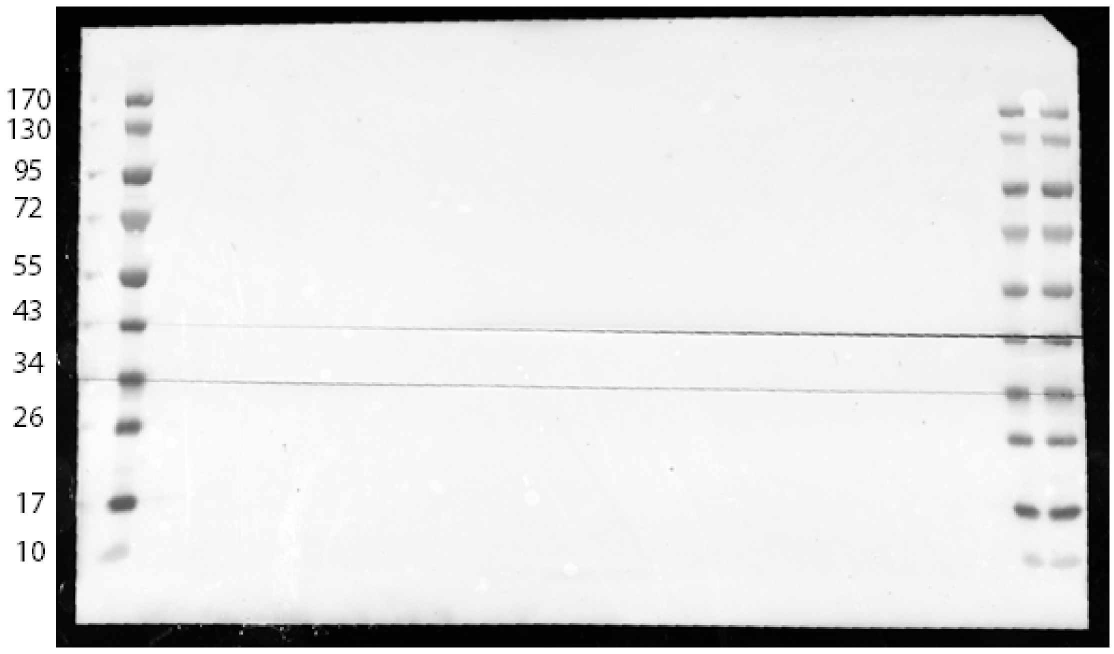

Supplement: Supplementary file 2 — Additional file 2. [file 12943_2024_1989_MOESM2_ESM.pdf]
