# Supplementary material for: NEMO/NF-κB signaling functions as a double-edged sword in PanIN formation versus progression to pancreatic cancer
Source: Mol Cancer. 2024 May 16;23:103. doi: 10.1186/s12943-024-01989-x (PMC11097402; doi:10.1186/s12943-024-01989-x)
Supplement: Supplementary file 3 — Additional file 3. [file 12943_2024_1989_MOESM3_ESM.docx]

**SUPPLEMENTARY FIGURES AND TABLES**

**
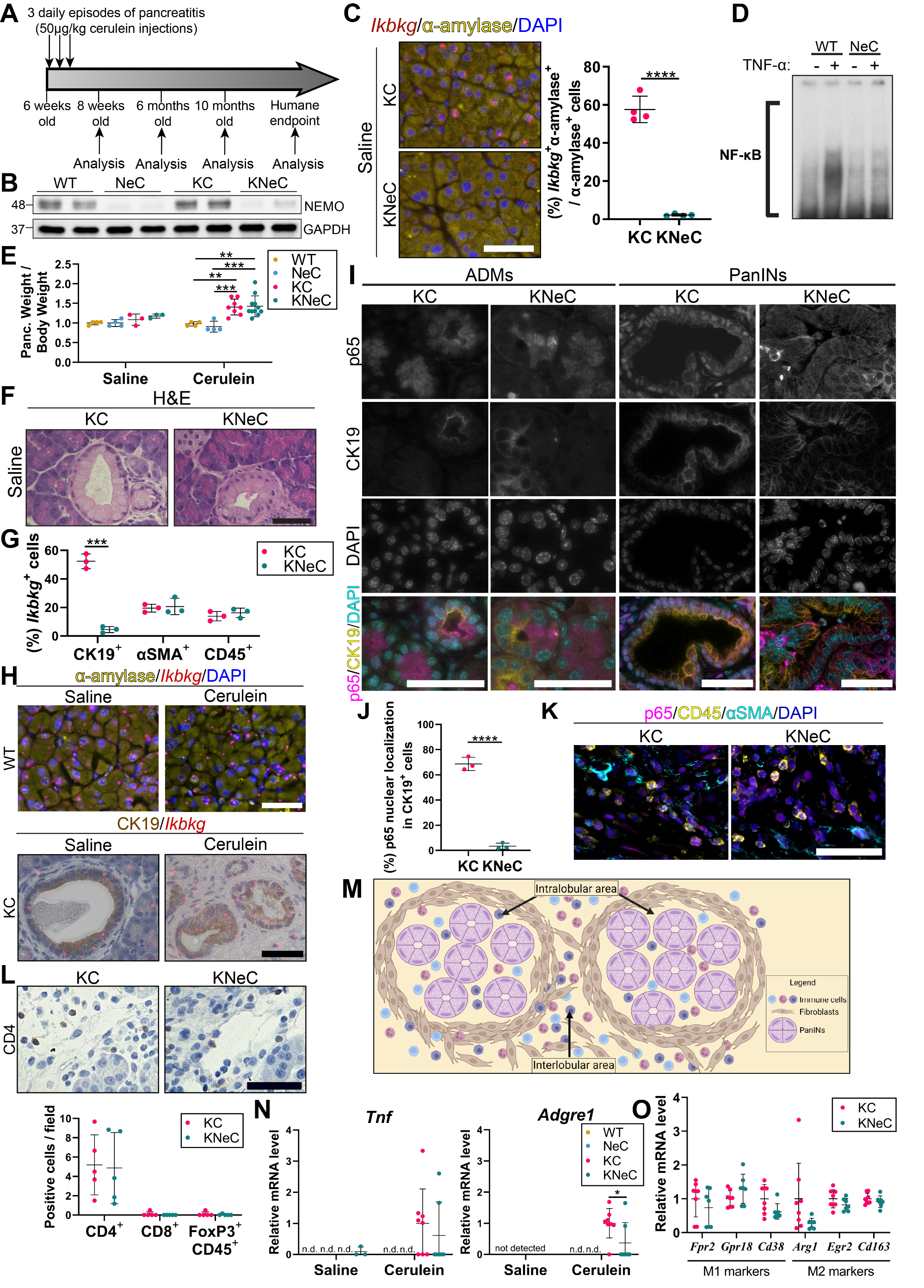
**

**Supplementary Figure S1**: NEMO ablation effect in different assays at the age of 8 weeks. **(A)** Mice were subjected to three daily episodes of cerulein injections. Additional information is provided in material and methods section. **(B)** Western blot analysis of pancreatic protein extracts from wild-type (WT), Pdx1-Cre;NEMO^fl/fl^ (NeC), Pdx1-Cre;LSL-KRAS^G12D^ (KC) and Pdx1-Cre;LSL-KRAS^G12D^;NEMO^fl/fl^ (KNeC) mice. GAPDH was used as a loading control. T=8 weeks. **(C)** Left: Visualization of *Ikbkg* transcripts in α-amylase^+^ cells in saline-injected KC and KNeC mice. Scalebar: 50μm. Right: Quantification of *Ikbkg^+^*α-amylase^+^/α-amylase^+^ cells. N=4 mice/group; t=8 weeks. Two-tailed Student’s t test. **(D)** EMSA of nuclear extracts from acinar cells isolated from WT and NeC mice using a radiolabeled double-stranded DNA probe containing an Ig-κ enhancer consensus NF-κB site. **(E)** Quantification of pancreatic weight to body weight ratio of saline- or cerulein-injected WT, NeC, KC and KNeC mice. Cerulein-injected KC and KNeC groups: N≥7 mice/group, rest of the groups: N≥3 mice/group; t=8 weeks. One-way ANOVA-Tukey for saline-injected groups; One-way ANOVA-Tukey for cerulein-injected groups. **(F)** Visualization of low-grade PanINs in saline-injected KC and KNeC mice. Scalebar: 50μm; t=8 weeks. **(G)** Percentage of *Ikbkg*^+^CK19^+^/CK19^+^ cells, *Ikbkg*^+^αSMA^+^ /αSMA^+^ cells and *Ikbkg*^+^CD45^+^/CD45^+^ cells of pancreata of cerulein-injected mice. N=3 mice/group; t=8 weeks. Two-tailed Student’s t test. **(H)** Visualization of *Ikbkg* transcripts in (Top) acinar cells and (Bottom) lesions of saline- or cerulein-injected WT and KC mice. Scalebars: 50μm; t=8 weeks. **(I)** Staining of pancreata of cerulein-injected KC and KNeC mice against p65 and CK19. Scalebars: 50μm; t=8 weeks. **(J)** Percentage of CK19^+^ cells with p65 nuclear localization. N=3 mice/group; t=8 weeks. Two-tailed Student’s t test.  **(K)** Staining of pancreata of cerulein-injected KC and KNeC mice against p65, αSMA and CD45. Scalebar: 50μm; t=8 weeks. **(L)** Top: Visualization of CD4^+^ cells on pancreata of cerulein-injected KC and KNeC mice. Scalebar: 50μm. Bottom: Quantification of CD4^+^, CD8^+^ and FoxP3^+^CD45^+^ cells. N=5 mice/group; t=8 weeks. Two-tailed Student’s t test. **(M)** Graphical representation of murine pancreas with pancreatic lesions indicating the intralobular and the interlobular area. **(N)** qRT-PCR in pancreatic tissue of saline- or cerulein-injected mice. The results are given relative to cerulein-injected KC mice, which were set to 1. Cerulein-injected KC and KNeC groups: N≥7 mice/group, rest of the groups: N≥3 mice/group; t=8 weeks, n.d. = not detected. One-way ANOVA-Tukey for saline-injected groups; One-way ANOVA-Tukey for cerulein-injected groups. **(O)** qRT-PCR in pancreatic tissue of cerulein-injected KC and KNeC mice. The results are given relative to cerulein-injected KC mice, which were set to 1. N≥7 mice/group; t=8 weeks. Two-tailed Student’s t test.

Dot plot: Dots represent individual animals. n.s.: p > 0.05; *p < 0.05; **p < 0.01; ***p < 0.001; ****p < 0.0001.

**
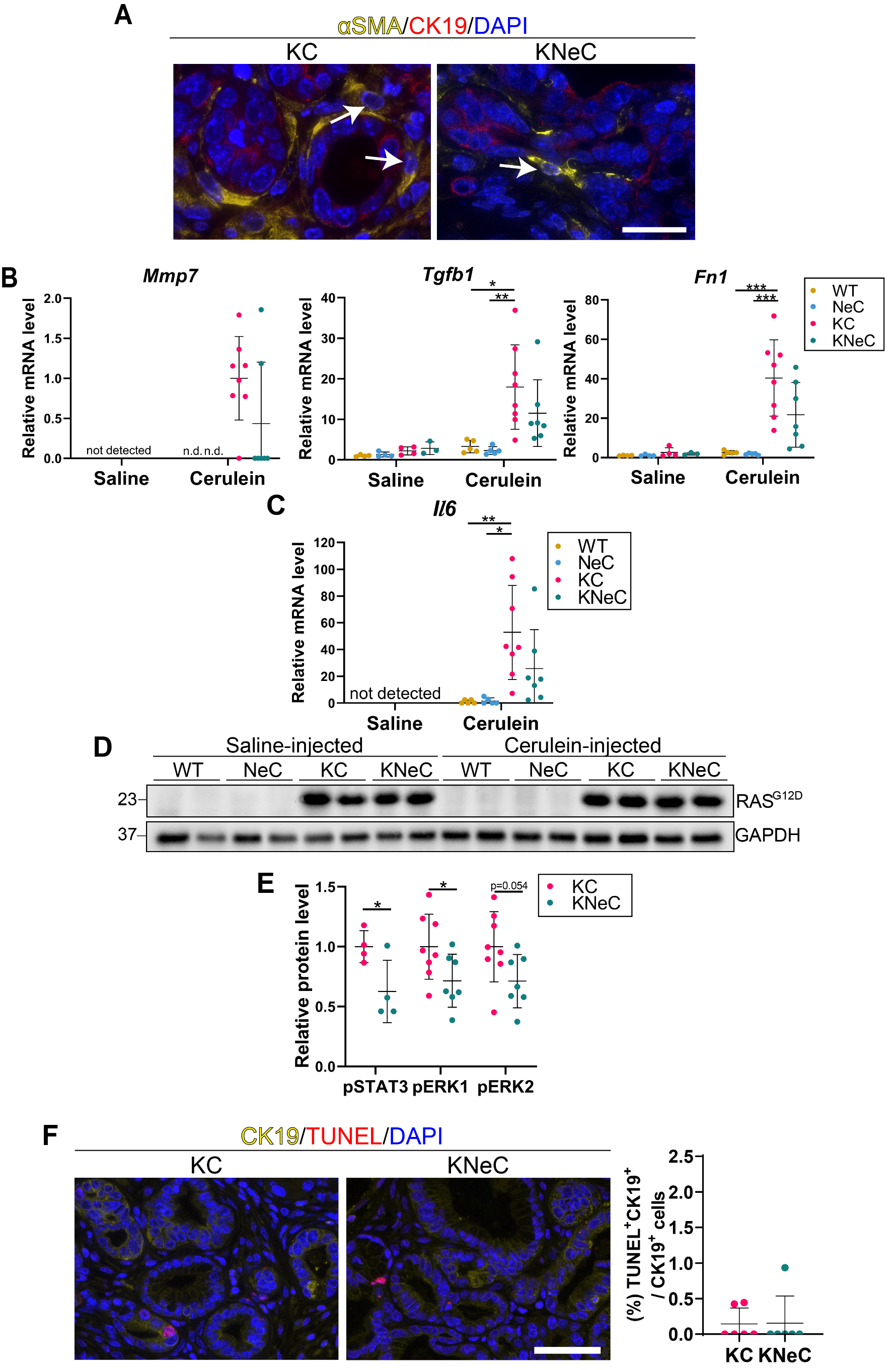
**

**Supplementary Figure S2**: Quantification of RNA transcripts and protein levels from pancreata of 8-week-old mice. **(A)** High magnification of αSMA^+^ cells in pancreata of cerulein-injected 8-week-old KC and KNeC mice. Scalebar: 25μm. (B) Quantitative RT-PCR for the expression of the indicated transcripts in pancreatic tissue of saline- or cerulein-injected 8-week-old mice, given relative to saline-injected wild-type mice for *Tgfb1* and *Fn1,* and to cerulein-injected KC mice for *Mmp7*, which were set to 1. Cerulein-injected KC and KNeC groups: N≥7 mice/group, rest of the groups: N≥3 mice/group; t=8 weeks. One-way ANOVA-Tukey for saline-injected groups; One-way ANOVA-Tukey for cerulein-injected groups. **(C)** Quantitative RT-PCR for the expression of the indicated transcript in pancreatic tissue of saline- or cerulein-injected mice, given relative to cerulein-injected wild-type mice, which were set to 1. Cerulein-injected KC and KNeC groups: N≥7 mice/group, rest of the groups: N≥3 mice/group; t=8 weeks. One-way ANOVA-Tukey for saline-injected groups; One-way ANOVA-Tukey for cerulein-injected groups. **(D)** Western blot analysis of pancreatic protein extracts from WT, NeC, KC and KNeC mice for the indicated protein. GAPDH was used as a loading control. T=8 weeks. **(E)** Quantification of western blot analysis for the cerulein-injected KC and KNeC mice. The diagrams show the quantification of the pERK1/ERK1, pERK2/ERK2 and pSTAT3/STAT3 ratios, given relative to cerulein-injected KC mice, which were set to 1. N≥4/group; t=8 weeks. Two-tailed Student’s t test. **(F)** Left: Visualization of CK19^+^TUNEL^+^ cells in cerulein-injected 8-week-old mice. Scalebar: 50μm. Right: Quantification of CK19^+^TUNEL^+^/CK19^+^ cells. N=6 mice/group; t=8 weeks. Two-tailed Student’s t test.

Dot plot: Dots represent individual animals. n.s.: p > 0.05; *p < 0.05; **p < 0.01; ***p < 0.001.


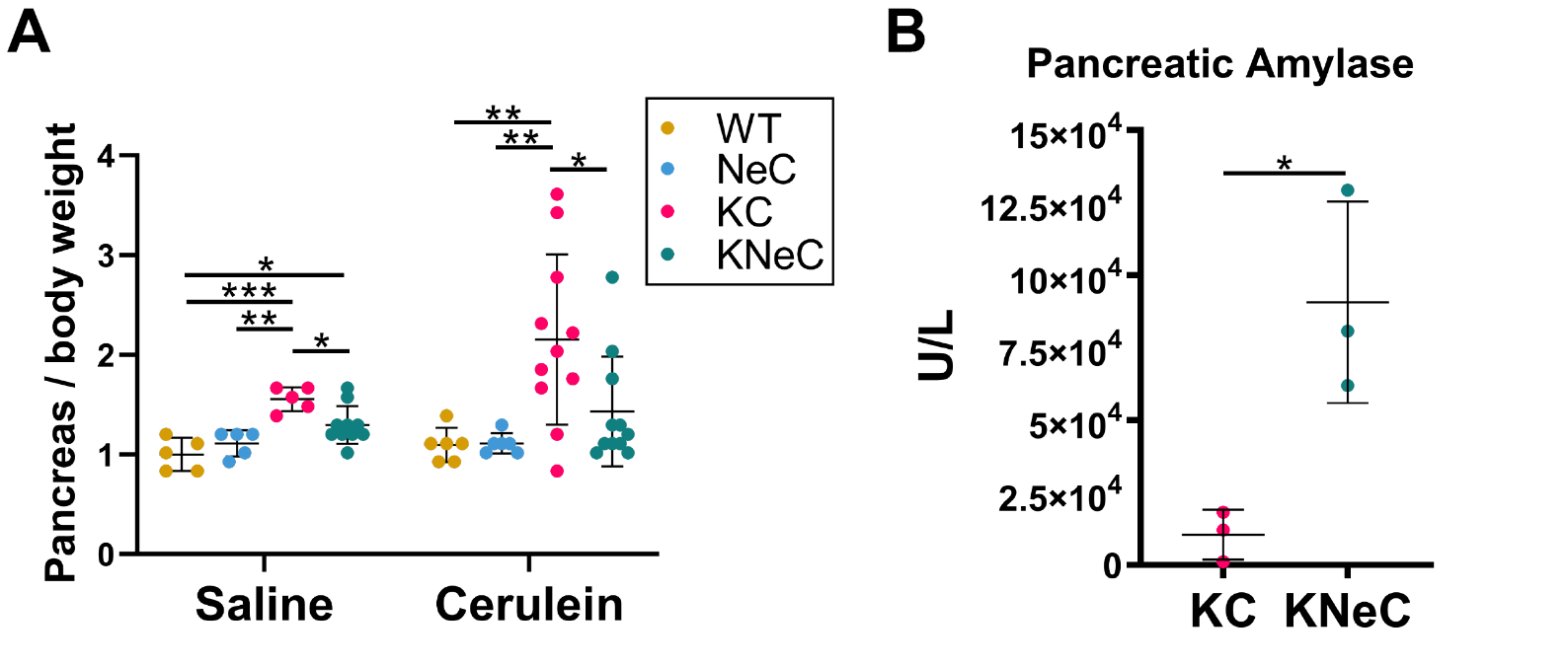


**Supplementary Figure S3**: Quantification of pancreatic weight and amylase activity of total pancreas of 10-month-old mice. **(A)** Quantification of pancreatic weight to body weight ratio of saline- or cerulein-injected WT, NeC, KC and KNeC mice. Cerulein-injected KC and KNeC groups: N≥7 mice/group, rest of the groups: N≥3 mice/group; t=10 months). One-way ANOVA-Tukey for saline-injected groups; One-way ANOVA-Tukey for cerulein-injected groups. **(B)** Pancreatic amylase enzymatic activity of pancreas as measured in a solution of 1μg pancreas/μl in pancreata of cerulein-injected KC and KNeC mice. N≥3 mice/group; t=10 months. Two-tailed Student’s t test**.**

Dot plot: Dots represent individual animals. n.s.: p > 0.05; *p < 0.05; **p < 0.01; ***p < 0.001.

**
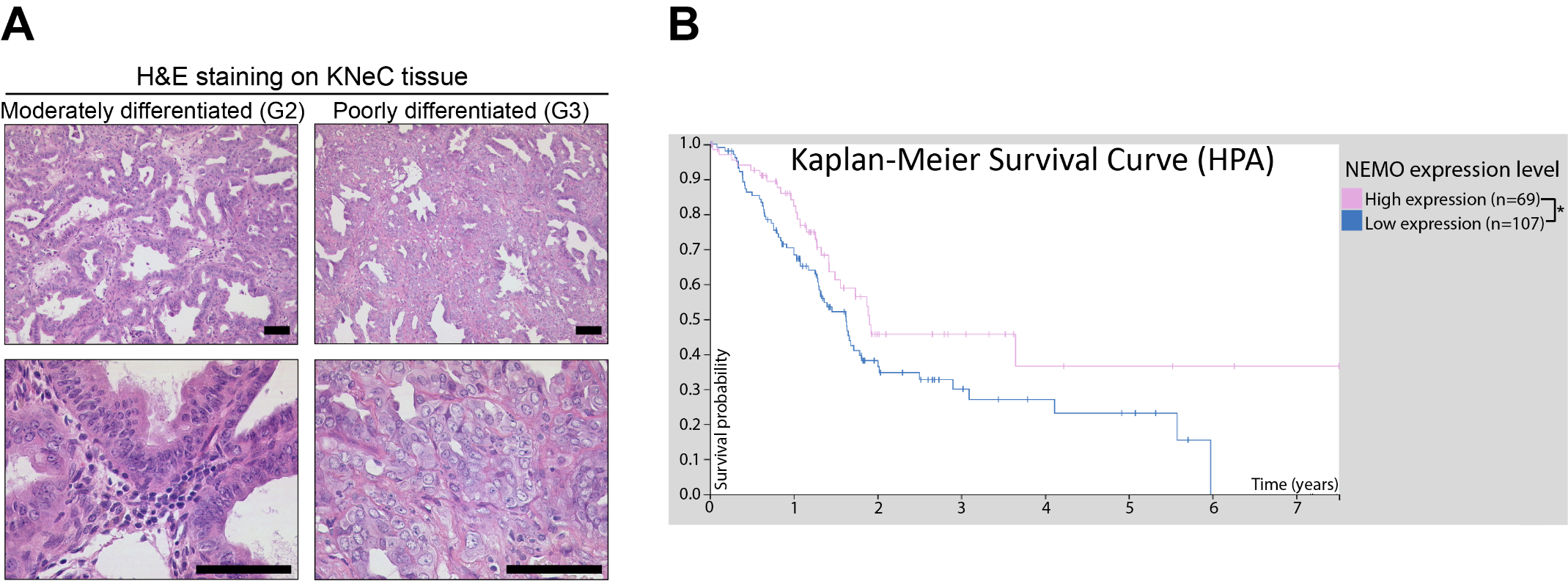
**

**Supplementary Figure S4**: Examination of cerulein-injected KC and KNeC mice at the time point of 10 months or at their humane end point (HEP). **(A)** Visualization of PDAC with moderately or poorly differentiated status deriving from cerulein-injected KNeC mice; scalebars: 50μm; t=10 months. **(B)** Kaplan-Meier survival curve for high expression (pink line) and low expression (blue line) of NEMO in pancreatic cancer patients. The graph is derived and modified from the human protein atlas website. Log-rank test.


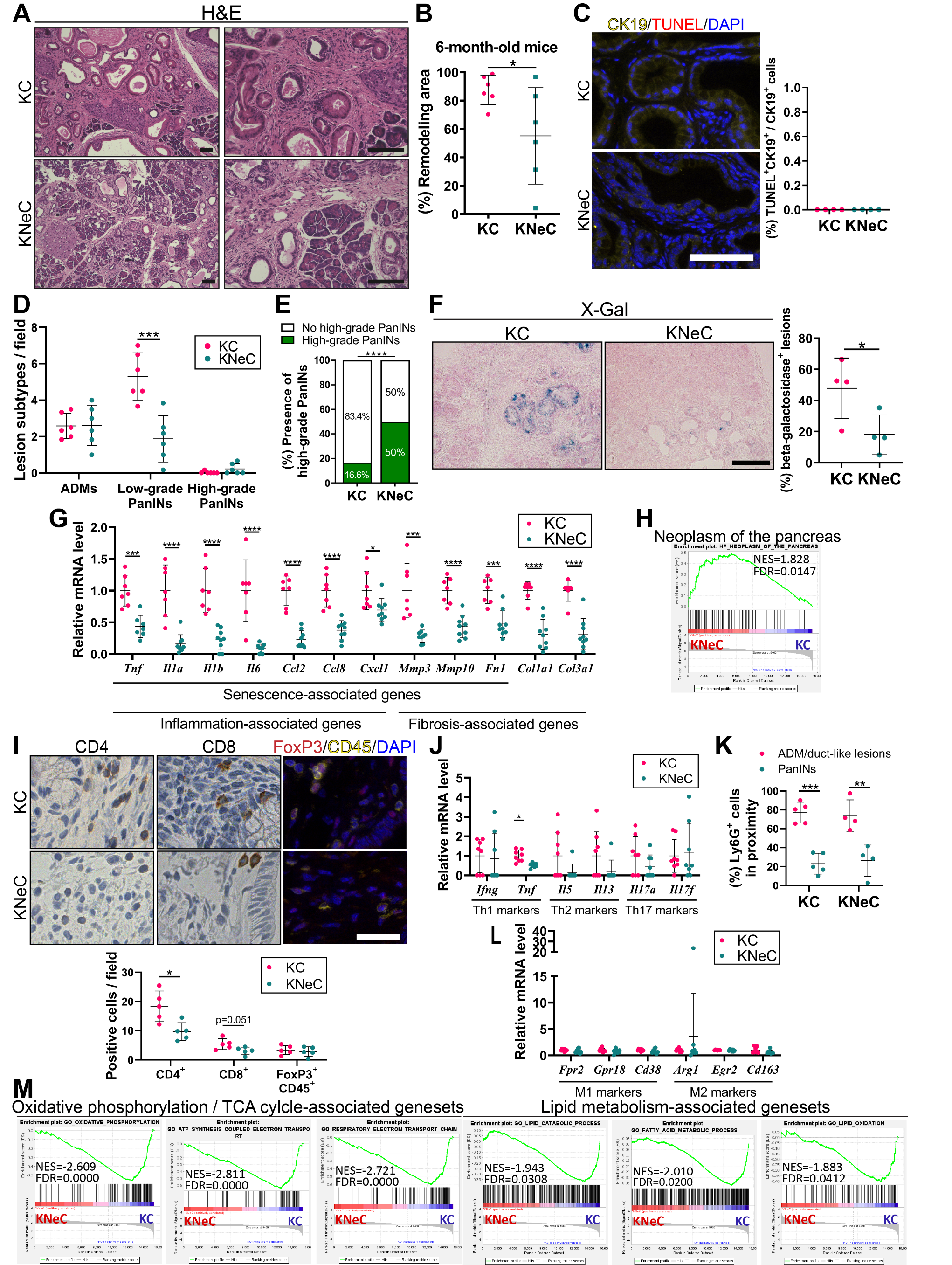


**Supplementary Figure S5**: Examination of pancreata of 6-month-old and 10-month-old cerulein-injected KC and KNeC mice. **(A)** H&E staining of pancreatic tissue from cerulein-injected KC and KNeC mice (t=6 months) on low and high magnifications. Scalebars=100μm. **(B)** Percentage of the remodeling area (neoplasia, cancer, fibrosis, inflammation) to the total pancreatic area on pancreatic sections of cerulein-injected KC and KNeC mice. N=6 mice/group; t=6 months. Two-tailed Student’s t test. **(C)** Left: Visualization of CK19^+^TUNEL^+^ cells in cerulein-injected mice. Scalebar: 50μm. Right: Quantification of CK19^+^TUNEL^+^/CK19^+^ cells. N=4 mice/group; t=6 months. Two-tailed Student’s t test. **(D)** Quantification of ADMs, low-grade and high-grade PanINs on pancreatic sections of cerulein-injected KC and KNeC mice. N=6 mice/group; t=6 months. Two-tailed Student’s t test**. (E)** Percentage of high-grade PanIN presence in cerulein-injected KC and KNeC mice. N=6 mice/group; t=6 months. Two-tailed Fisher’s exact test. **(F)** Left: X-gal staining on pancreatic tissue of cerulein-injected KC and KNeC mice. Scalebar: 100 μm. Right: Quantification of beta-galactosidase^+^ lesions to the total lesions. N=4 mice/group; t=6 months. Two-tailed Student’s t test. **(G)** Quantitative RT-PCR for the expression of the indicated transcripts in pancreatic tissue of cerulein-injected mice, given relative to KC mice, which were set to 1. N≥7 mice/group; t=6 months. Two-tailed Student’s t test. **(H)** GSEA of cerulein-injected KC and KNeC mice for the neoplasm of the pancreas geneset. N=3 mice/group, t=10 months. **(I)** Top: Visualization of CD4^+^, Cd8^+^ and FoxP3^+^CD45^+^ cells on pancreatic sections of cerulein-injected KC and KNeC mice. Scalebar: 25μm. Bottom: Quantification of CD4^+^, Cd8^+^ and FoxP3^+^CD45^+^ cells. N=5 mice/group; t=10 months. Two-tailed Student’s t test. **(J)** Quantitative RT-PCR for the expression of the indicated Th1-, Th2- and Th17-associated transcripts in pancreatic tissue of cerulein-injected KC and KNeC mice. The results are given relative to cerulein-injected KC mice, which were set to 1. N≥7 mice/group; t=10 months. Two-tailed Student’s t test. **(K)** Percentage of Ly6G^+^ cells being closer to either ADM/duct-like lesion or PanIN in a maximum radius of 20μm. N≥5 mice/group; t=10 months. Two-tailed Student’s t test. **(L)** Quantitative RT-PCR for the expression of the indicated M1- and M2-asosciated transcripts in pancreatic tissue of cerulein-injected KC and KNeC mice. The results are given relative to cerulein-injected KC mice, which were set to 1. N≥7 mice/group; t=10 months. Two-tailed Student’s t test. **(M)** GSEA of cerulein-injected KC and KNeC mice for oxidative phosphorylation-, TCA cycle- and lipid metabolism-associated genesets. N=3 mice/group; t=10 months.

Dot plot: Dots represent individual animals. n.s.: p > 0.05; *p < 0.05; **p < 0.01; ***p < 0.001; ****p < 0.0001.


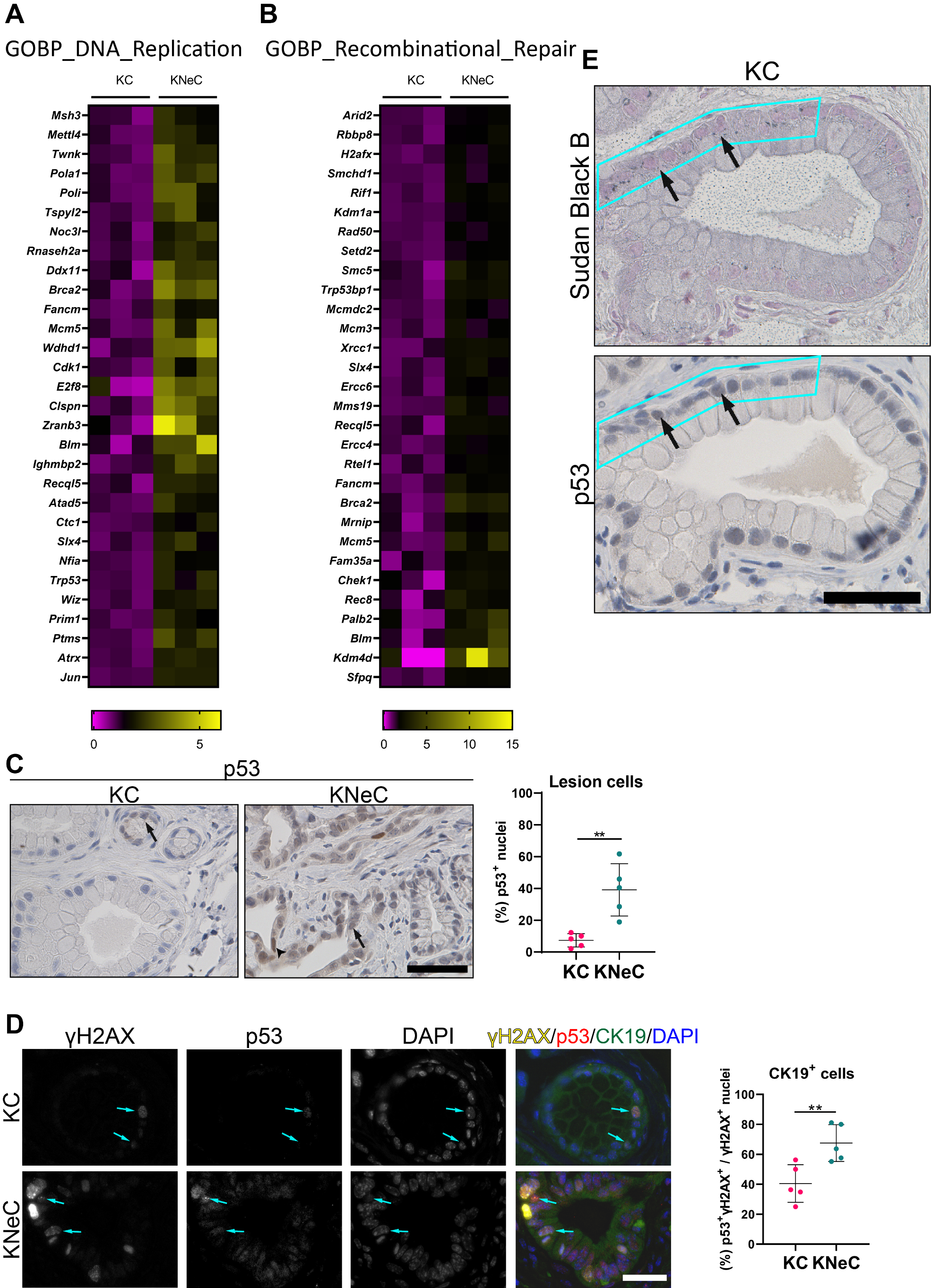


**Supplementary Figure S6: (A)** GO_BP DNA Replication heatmap of pancreatic lesions of cerulein-injected KC and KNeC mice presenting the relative level of each transcript. N=3 mice/group; t=10 months. **(B)** GO_BP Recombinational Repair heatmap of pancreatic lesions of cerulein-injected KC and KNeC mice presenting the relative level of each transcript. N=3 mice/group; t=10 months. **(C)** Left: Immunohistochemical analysis of p53 on pancreatic sections of 10-month-old cerulein-injected KC and KNeC mice. Arrow: Weakly p53-stained nucleus; Arrowhead: Strongly p53-stained nucleus. Scalebar: 50μm. Right: Percentage of p53^+^ lesion cells. N=5 mice/group; t=10 months. Two-tailed Student’s t test. **(D)** Left: Visualization of γH2AX^+^, p53^+^ and CK19^+^ cells on pancreatic sections of 10-month-old cerulein-injected KC and KNeC mice. Arrows: γH2AX^+^ nuclei. Scalebar: 25μm. Right: Quantification of p53^+^γH2AX^+^CK19^+^ cells to total γH2AX^+^CK19^+^ cell number. N=5 mice/group; t=10 months. Two-tailed Student’s t test. **(E)** Top: SBB staining on pancreatic serial section of 10-month-old cerulein-injected KC mouse. Perinuclear black spots indicate positive cells. Bottom: p53 staining on pancreatic serial section of 10-month-old cerulein-injected KC mouse. Cyan-marked area indicates cluster of SBB-positive cells. Arrow: p53-stained nucleus. Scalebar: 50μm.

Dot plot: Dots represent individual animals. n.s.: p > 0.05; *p < 0.05; **p < 0.01.

**Supplementary Table S1.** Number of captured fields per staining for statistical analysis.

| **Type of staining** | **Number of fields** | |
| --- | --- | --- |
| H&E staining for lesion number counting | | 8 random fields  Whole section  6 random fields  6 random fields  6 random fields  6 random fields  6 random fields  6 random fields  6 random fields  6 random fields  6 random fields  6 random fields  6 random fields  6 random fields  6 random fields  > 100 cells (Recombination rate)  > 100 cells (NF-κB localization)  6 random fields  6 random fields  6 random fields  6 random fields  6 random fields 6 random fields  6 random fields  6 random fields  6 random fields |
| H&E staining for total remodeling area  CD45/DAPI staining  F4/80 staining  CD3 staining  Ly6G staining  B220 staining  CD3 staining  CD8 staining  FoxP3/CD45/DAPI staining  aSMA/CK19/DAPI staining  Azan Trichrome staining  Xgal staining  SBB staining  α-amylase/CK19/DAPI staining  *Ikbkg*/α-amylase/DAPI staining  p65/CK19/DAPI staining  *Ikbkg*/CK19 staining  *Ikbkg*/αSMA/DAPI staining  *Ikbkg*/CD45/DAPI staining  Ki67/γH2AX/CK19/DAPI staining  TUNEL/CK19 staining p53/γH2AX/CK19/DAPI staining  Ki67 staining (Brightfield)  p53 staining (Brightfield)  γH2AX/DAPI staining (cell culture) | |  |

**Supplementary Table S2.** Primers and primary antibodies used in the study.

| **Gene** | **Forward primer sequence** | **Reverse primer sequence** |
| --- | --- | --- |
| *Adgre1*  *Arg1*  *Ccl2*  *Ccl8*  *Cd163*  *Cd38*  *Cxcl1*  *Col1α1* | ggaggacttctccaagcctatt  tgcgccacatgaaaaccatc  catccacgtgttggctca  ttctttgcctgctgctcata  atgacctggcatgcaatgga  cgacatcgaaggagcttcca  cagagcctctaaccagttcca  catgttcagctttgtggacct | aggcctctcagacttctgctt  caagcccttgggaggagaag  gatcatcttgctggtgaatgagt  gcaggtgactggagccttat  agttatgcttgccccatccc  ggcctgtagttatccacgca  tgggatcatggtgctgtg  gcagctgacttcagggatgt |
| *Col3α1*  *Egr2*  *Fn1*  *Fpr2*  *Gpr18*  *Ifng*  *Il1a*  *Il1b*  *Il5*  *Il6*  *Il13*  *Il17a*  *Il17f*  *Mmp3*  *Mmp7*  *Mmp10*  *Tgfb1*  *Tnf* | tcccctggaatctgtgaatc  catgcagtgagtggtgttgc  gatgccgatcagaagtttgg  tgttctgcatccagtctggg  taacacaggcgactctgagc  atctggaggaactggcaaaa  ttggttaaatgacctgcaaca  agttgacggaccccaaaag  acattgaccgccaaaaagag  gctaccaaactggatataatcagga  gtgtctctccctctgaccct  ggaaagctggaccaccacat  cttgcagaaggctgggaact  tgcagctctactttgttctttga  ttctgctttgtgtgtctgctg  tggattctgccattgagaaag  tggagcaacatgtggaactc  tgcctatgtctcagcctcttc | tgagtcgaattggggagaat  gaccctgttaacactgccca  ggttgtgcagatctcctcgt  aaatccagggcccaacaacc  tctgagaggtgaccgtttgc  ttcaagacttcaaagagtctgagg  gagcgctcacgaacagttg  agctggatgctctcatcagg  atccaggaactgcctcgtc  ccaggtagctatggtactccagaa  ggggagtctggtcttgtgtg  ctcaggctccctcttcagga  gggacagaaatgccctggtt  agagatttgcgccaaaagtg  ccttctttgttttagagtcatgagg  ggtaaaagtctccgtgttctcc  gtcagcagccggttacca  gaggccatttgggaacttct |

| **Western Blot: Primary antibodies** | | | | |
| --- | --- | --- | --- | --- |
| **Antibody** | **Company** | **Catalog number** | **Dilution** | **Host Species** |
| Erk2 | Santa Cruz | sc-154 | 1:1000 | rabbit |
| GAPDH  NEMO/IKKγ  Phospho-ERK1/2  Phospho-STAT3  RasG12D | Santa Cruz  BD Biosciences  Cell Signaling  Cell Signaling  Cell Signaling | sc-25778  611306  4370  9145S  14429S | 1:1000  1:1000  1:1000  1:1000  1:1000 | rabbit  mouse  rabbit  rabbit  rabbit |
| STAT3 | Cell Signaling | 4904S | 1:1000 | rabbit |

| **Immunostaining: Primary antibodies** | | | | |
| --- | --- | --- | --- | --- |
| **Antibody** | **Company** | **Catalog number** | **Dilution** | **Host Species** |
| α-amylase  α-SMA  B220  CD3  CD4  CD8  CD45 | MilliporeSigma  MilliporeSigma  Pharmigen  ThermoFischer  Abcam  Abcam  BD Biosciences | A8273  CBL171  553084  MA1-90582  ab183685  ab217344  550539 | 1:400  1:100  1:4000  1:300  1:100  1:100  1:100 | rabbit  mouse  rat  rabbit  rabbit  rabbit  rat |
| CK19  CK19  CK7  FoxP3  F4/80  Ki67  Ki67  Ly6G  p53  p65  γH2AX  γH2AX | MilliporeSigma  Santa Cruz  DAKO  R&D  Biomedicals AG  Biolegend  ThermoFischer  Pharmigen  Abcam  Neomarkers  Novus Biological  Cell Signaling | MABT913  sc-33111  GA619  MAB8214  T-2006  652402  MA515420  551459  ab241566  RB1638  NB100-384  cs-9718 | 1:150  1:100  ready to use  1:100  1:50  1:100  1:100  1:600  1:100  1:100  1:1000  1:100 | rat  goat  mouse  rabbit  rat  rat  rabbit  rat  rat  rabbit  rabbit  rabbit |

**Supplementary Table S3**: Cytokines detected in C3, C4 membranes. OIS-associated cytokines are in bold characters.

| **N** | **Eotaxin** | **Eotaxin** | IL3 Rb | IL3 Rb | L-selectin | L-selectin | SCF | SCF | POS | POS |  |  |  |  |  |  |  |  |  |  |
| --- | --- | --- | --- | --- | --- | --- | --- | --- | --- | --- | --- | --- | --- | --- | --- | --- | --- | --- | --- | --- |
| **M** | CXCL16 | CXCL16 | IL3 | IL3 | **LIX** | **LIX** | RANTES | RANTES | Blank | Blank |  |  |  |  |  |  |  |  |  |  |
| **L** | CTACK | CTACK | IL2 | IL2 | Leptin | Leptin | P-Selectin | P-Selectin | Blank | Blank |  | **L** | GITR | GITR | MMP-2 | MMP-2 | VEGF R1 | VEGF R1 | POS | POS |
| **K** | CRG-2 | CRG-2 | **IL1-beta** | **IL1-beta** | Leptin R | Leptin R | PF4 | PF4 | VEGF | VEGF |  | **K** | Flt-3 Ligan | Flt-3 Ligan | MDC | MDC | TSLP | TSLP | Blank | Blank |
| **J** | CD40 | CD40 | **IL1-alpha** | **IL1-alpha** | **KC** | **KC** | MIP-3-alpha | MIP-3-alpha | VCAM-1 | VCAM-1 |  | **J** | Fcg RIIB | Fcg RIIB | Lungkine | Lungkine | TROY | TROY | Blank | Blank |
| **I** | TNFRSF8 | TNFRSF8 | IGF-BP-6 | IGF-BP-6 | IL17 | IL17 | MIP-3-beta | MIP-3-beta | TPO | TPO |  | **I** | E-Selectin | E-Selectin | I-TAC | I-TAC | TRANCE | TRANCE | Blank | Blank |
| **H** | CD30L | CD30L | IGF-BP-5 | IGF-BP-5 | **IL13** | **IL13** | MIP-2 | MIP-2 | sTNF RII | sTNF RII |  | **H** | Dtk | Dtk | **IL-7** | **IL-7** | TIMP-2 | TIMP-2 | Blank | Blank |
| **G** | **BLC** | **BLC** | IGF-BP-3 | IGF-BP-3 | IL12-p70 | IL12-p70 | MIP-1-gamma | MIP-1-gamma | sTNF RI | sTNF RI |  | **G** | DPPIV/CD26 | DPPIV/CD26 | IL-17B R | IL-17B R | Thymus CK-1 | Thymus CK-1 | Blank | Blank |
| **F** | axl | axl | **IFN-gamma** | **IFN-gamma** | IL12-p40/p70 | IL12-p40/p70 | **MIP-1-alpha** | **MIP-1-alpha** | TNF-alpha | TNF-alpha |  | **F** | **bFGF** | **bFGF** | **IL-15** | **IL-15** | Shh-N | Shh-N | Blank | Blank |
| **E** | Blank | Blank | **GM-CSF** | **GM-CSF** | IL10 | IL10 | MIG | MIG | TIMP-1 | TIMP-1 |  | **E** | Blank | Blank | IGF-II | IGF-II | Resistin | Resistin | Blank | Blank |
| **D** | NEG | NEG | G-CSF | G-CSF | IL9 | IL9 | **M-CSF** | **M-CSF** | **TECK** | **TECK** |  | **D** | NEG | NEG | IGF-I | IGF-I | Pro-MMP-9 | Pro-MMP-9 | Blank | Blank |
| **C** | NEG | NEG | Fractalkine | Fractalkine | **IL6** | **IL6** | MCP-5 | MCP-5 | **TCA-3** | **TCA-3** |  | **C** | NEG | NEG | **IGFBP-2** | **IGFBP-2** | **Osteoporotegerin** | **Osteoporotegerin** | VEGF-D | VEGF-D |
| **B** | POS | POS | FAS ligand | FAS ligand | IL5 | IL5 | MCP-1 | MCP-1 | TARC | TARC |  | **B** | POS | POS | ICAM-1 | ICAM-1 | Osteopontin | Osteopontin | VEGF R3 | VEGF R3 |
| **A** | POS | POS | Eotaxin-2 | Eotaxin-2 | IL4 | IL4 | Lymphotactin | Lymphotactin | **SDF-1-alpha** | **SDF-1-alpha** |  | **A** | POS | POS | HGF R | HGF R | **MMP-3** | **MMP-3** | VEGF R2 | VEGF R2 |
| **C3** | **1** | **2** | **3** | **4** | **5** | **6** | **7** | **8** | **9** | **10** |  | **C4** | **1** | **2** | **3** | **4** | **5** | **6** | **9** | **10** |
